# Innate immune dysfunction and persistent activation in South African HIV elite controllers

**DOI:** 10.3389/fimmu.2025.1603436

**Published:** 2025-08-27

**Authors:** Asisipo Mohamed, Yenzekile Zungu, Sharon Shalekoff, Osman Ebrahim, Ziyaad Waja, Neil Martinson, Caroline T. Tiemessen, Christina Thobakgale

**Affiliations:** ^1^ School of Pathology, Faculty of Health Sciences, University of the Witwatersrand, Johannesburg, South Africa; ^2^ Centre for HIV and STIs, National Institute for Communicable Diseases, Division of the National Health Laboratory Service, Faculty of Health Sciences, University of the Witwatersrand, Johannesburg, South Africa; ^3^ School of Therapeutic Sciences, Department of Pharmacology, Faculty of Health Sciences, University of the Witwatersrand, Johannesburg, South Africa; ^4^ Perinatal HIV Research Unit, Chris Hani Baragwanath Academic Hospital, Soweto, University of the Witwatersrand, Johannesburg,, South Africa

**Keywords:** HIV-1, HIV elite controllers, antigen-presenting cells, monocyte activation, proinflammatory cytokines

## Abstract

**Background:**

Elite controllers can spontaneously control HIV-1 infection without antiretroviral treatment but remain at risk of developing non-AIDS-related conditions. The adaptive immune system is key in mediating spontaneous viral control; however, the innate immune response remains understudied. We assessed the quality of the innate immune responses by evaluating the phenotype and function of antigen-presenting cells (APCs) in South African adults living with HIV (PWH).

**Methodology:**

A total of 73 black South Africans were included in this study. Of these, 55 were living with HIV and included 16 individuals with spontaneous viral control (PWH_EC_), 20 HIV progressors (PWH_PROG_), and 19 individuals suppressed on ART (PWH_ART_). Eighteen individuals without HIV infection (PWOH_HIV-_) served as the control group. Monocyte subsets, T cell and monocyte activation and the production of tumour necrosis factor-alpha (TNF-α), interferon-alpha (IFN-α), and interleukin-1 beta (IL-1β) by monocytes, myeloid (mDCs) and plasmacytoid (pDCs) dendritic cells were analyzed using multicolour flow cytometry following stimulation with toll-like receptor (TLR)4 (LPS), TLR7/8 (CL097), and TLR9 (CpG-ODN2216) ligands. Plasma biomarkers, soluble CD14 (sCD14), and D-dimer were assessed using enzyme-linked immunosorbent assay.

**Results:**

Our findings show a reduced expression of CD86 on monocytes of PWH_EC_ (p=0.04) compared to PWOH_HIV-_. A reduced frequency of the classical monocyte (CD14+CD16) subset in PWH_EC_ (p=0.02) and PWH_PROG_ (p=0.05) compared to PWOH_HIV-_. TNF-α and IL-1β production was lower in monocytes and mDCs of PWH_EC_ compared to PWOH_HIV-_ post-stimulation with TLR4, and TLR7/8 (all p<0.05). Increased sCD14 levels in PWH_EC_ compared to PWOH_HIV-_ (p=0.01) indicate persistent immune activation, whereas increased D-dimer levels in PWH_PROG_ compared to PWH_ART_ (p=0.01) and PWH_EC_ (p=0.04) suggest higher inflammation in PWH_PROG_.

**Conclusion:**

PWH_EC_ exhibits similar immune responses as other PWH including PWH_PROG_, their innate immune profiles are characterized by lower levels of monocyte activation, reduced levels of classical monocytes, reduced capacity to produce pro-inflammatory cytokines, and elevated biomarkers associated with unfavourable disease outcomes. These findings highlight the need for continuous monitoring and potential therapeutic interventions to mitigate chronic inflammation in PWH_EC_. Furthermore, it expands our understanding of complex innate immune cell responses in PWH_EC_.

## Introduction

The HIV/AIDS epidemic remains a global health crisis with approximately 39.9 million people living with HIV (PWH) worldwide. South Africa remains the epicentre of HIV-1 infection in sub-Saharan Africa with 7.8 million PWH in 2023 (1). Antiretroviral therapy (ART) has dramatically changed the prognosis of HIV infection and is widely available with 77% of South African PWH being on ART and suppressed by the end of 2023. ART suppresses HIV-1 replication to undetectable levels, significantly improving the lives of PWH by decreasing mortality and morbidity (2). However, ART has limitations including incomplete eradication of HIV-1 due to the persistence of viral reservoirs, which are the source of viral rebound if ART is discontinued (3). Despite ART, chronic inflammation, ongoing HIV-1 replication, and cellular metabolic dysregulation persist, contributing to non-infectious conditions like renal and cardiovascular diseases, as well as neurocognitive impairment, among others (4, 5).

Studies on natural HIV-1 control have identified a subgroup of PWH termed HIV controllers who naturally achieve viral control in the absence of ART for 2–10 years, maintain normal peripheral blood CD4+ T cell levels and have a low risk of progression to AIDS (6–8). HIV controllers are subdivided into viraemic controllers (VCs), long-term non-progressors (LTNPs) and elite controllers (ECs). Elite controllers can maintain viral control without disease progression for up to 25 years (9, 10). HIV-1-specific CD8+ T cells, especially those associated with HLA-I alleles B*27 and B*57, play a crucial role in the spontaneous control observed in elite controllers (10–12). Additionally, reduced expression of C-C chemokine receptor type 5 (CCR5) in elite controllers is correlated with slower disease progression due to reduced HIV viral entry. Genetic variations in the CCR5 gene are associated with an increased likelihood of spontaneous viral control (13). Furthermore, HLA class I alleles are genetic determinants that influence peptide presentation and cytotoxic T lymphocyte responses. However, less than 25% of elite controllers display these genetic phenotypes and variations, suggesting that other immunologic mechanisms are involved (11, 14). Studies have reported the role of T cell activation, cytotoxic T lymphocytes, and natural killer cells in spontaneous viral control, though the contribution of other innate immune cells remains less well understood (15).

Recent studies suggest that innate immune responses play a significant role in spontaneous HIV-1 control, and the concept of trained innate immune response suggests the possibility that spontaneous viral control will be achieved through the functions of innate immune cells (16–19). Marras et al. reported that increased interferon-gamma (IFN-γ) production and natural killer cells (NK cells) activation were linked to spontaneous viral control (3, 20). Previously, a study in our laboratory reported high levels of CD69-expressing NK cells in elite controllers which were associated with spontaneous viral control (19). The two major dendritic cell subsets, myeloid dendritic cells (mDCs) and plasmacytoid dendritic cells (pDCs) play distinct roles in HIV-1 control (3). mDCs, in elite controllers, display enhanced cGAS, IFN-α secretion, rapid maturation, HIV-1 viral sensing, effective antigen processing and subsequent CD4+ and CD8+ T cell activation (21). pDCs are the main producers of interferon-alpha (IFN-α) during an inflammatory response, however, this function is diminished in PWH, with reduced levels noted in viraemic controllers compared to elite controllers (11, 22–24).

Monocytes play a critical role in initiating the HIV-1 anti-viral inflammatory response by secreting inflammatory cytokines such as interleukin-6 (IL-6) and tumour necrosis factor-alpha (TNF-α) during the acute inflammatory phase of infection (25). Monocyte subsets are phenotypically and functionally distinct and can be classified into classical monocytes (CD14++/CD16-), intermediate monocytes (CD14++/CD16+), and non-classical monocytes (CD14+/CD16++) (26) according to the expression of CD14 (co-receptor for toll-like receptor 4) and CD16 (Fc gamma receptor IIIa) (27). During chronic HIV infection, intermediate monocytes expand and secrete pro-inflammatory cytokines, including TNF-α and IL-1β (27). Elite controllers exhibit reduced levels of intermediate monocytes compared to viraemic controllers, who have a higher viral load (28, 29). Non-classical monocytes express high levels of co-stimulatory markers CD80 and CD86, highlighting their possible role in antigen presentation during HIV-1 control in PWH with chronic/acute progressive infection (30–32). Furthermore, monocytes display reduced C-C chemokine receptor type 2 (CCR2) expression and elevated CX3CR1 expression in elite controllers (PWH_EC_) and people living with HIV suppressed on ART (PWH_ART_) compared to people without HIV (PWOH_HIV-_), highlighting the impact of HIV-1 on monocyte migration into tissues (3, 28). Together, these studies highlight the importance of innate immune cells in HIV antiviral immunity (26, 33).

Despite these insights, there is a substantial knowledge gap regarding the role of the innate immune response in HIV elite controllers. Furthermore, given their ability to maintain viral suppression without ART, elite controllers offer a unique opportunity to investigate natural mechanisms of HIV control. Understanding their innate immune responses may reveal therapeutic targets for achieving a functional cure. This study characterised monocyte subsets, their activation profiles compared to T cells, and the function of antigen-presenting cells (APCs) (monocytes, mDCs and pDCs) in different groups of South African PWH. Our findings show elevated T cell and reduced monocyte activation in South African PWH including PWH_EC_. In addition, a dysfunction in the ability of APCs to secrete pro-inflammatory cytokines (TNF-α, IFN-α and IL-1β) was observed in PWH compared to PWOH_HIV-_, together with increased plasma biomarkers associated with non-AIDS conditions.

## Materials and methods

### Study participants

This is a case-control study with different groups (different phenotypes) of people with HIV (PWH) and a negative control group. Study participants were Black adults (18 years and older). We recruited individuals who maintained spontaneous viral control, another group who had low CD4 counts and high viral loads at enrolment, and patients on long-term ART from Johannesburg at two sites: Parktown, and Soweto, South Africa (19). Finally, a group of controls without HIV infection (PWOH_HIV-_) were volunteers from the National Health Laboratory Services (NHLS), Sandringham Campus (19). The study participants were as follows; people living with HIV who maintained spontaneous viral control PWH_EC_ (n=16) with CD4 T cell count ≥500 cells/μl and viral load <50 copies/ml at enrolment, people living with HIV on ART for a minimum of 7 years PWH_ART_ (n=19) and virally suppressed at the time of enrolment with CD4 T cell count ≥500 cells/μl, people living with progressive HIV infection PWH_PROG_ (n=20) evidenced by their CD4 T cell count < 350 cells/mm^3^ (except n=4 HIV progressors where CD4 T cell count was above 350 cells/mm^3^) and viral load above> 5000 RNA copies/ml. A control group of people without HIV PWOH_HIV-_ (n=18) had a confirmed negative rapid HIV test and a negative plasma HIV ELISA assay at enrolment. People with HIV classified as progressors (PWH_PROG_) were not receiving antiretroviral therapy (ART) at the time of enrolment, allowing for the assessment of natural disease progression in the absence of treatment.

Informed consent was obtained from all the study participants. CD4 T cell count for all the participants was reported and formed part of the inclusion criteria. PWH_EC_ had undergone prior longitudinal clinical follow-ups of heterogeneous duration to ensure they are actual elite controllers maintaining high CD4 T cell levels and low viral load, not slow progressors. PWH_ART_ were virally suppressed for a minimum of 7 years at the time of enrolment, their CD4 T cell levels and viral load before ART commencement were not available. Blood samples from the respective participants were collected and cryopreserved at -150°C for later use. Ethical clearance was obtained from the University of the Witwatersrand Human Research Ethics Committee (Medical).

### PBMC isolation and thawing

T cell and monocyte activation, along with monocyte subsets, were assessed using cryopreserved peripheral blood mononuclear cells (PBMCs). PBMCs were isolated using ficoll density gradient centrifugation and immediately frozen. Cryopreserved PBMCs were thawed using a previously established protocol (34, 35). Briefly, PBMCs were resuspended in R10 medium (RPMI 1640 supplemented with 10% heat-inactivated foetal bovine serum (FBS), 1% 1000 U/ml penicillin, 1.7mM sodium glutamate, and 5.5ml HEPES), washed and rested in R10 medium (1x10^6^ cells/ml) at 37 ^°^C and 5% CO_2_ for approximately 2 hours before use in assays.

### Phenotypic staining of T cells and myeloid cell subsets

Peripheral blood mononuclear cells (1x10^6^ cells/ml) were stained with LIVE/DEAD Fixable Aqua Dead Cell Stain (Invitrogen, Carlsbad, California, USA) followed by surface staining with a monoclonal antibody cocktail including: CD56-BV510 (1H11), CD19-BV510 (HIB19), CD3-BV650 (OKT3), CD4-APC (OKT4), CD14-APC-CY7 (63D3), CD11c-PE-CY5 (3.9), CD16-BV786 (3G8), CD123-BV421 (6H6), CD69-Percp cy5.5 (FN50), CD38-PE (HIT2), and CD86-PECY-7 (IT2.2) all from (BioLegend, San Jose, USA), CD8-FITC (HIT8a), HLA-DR-PECF594 (L243) Becton Dickson and company (BD Biosciences, San Jose, USA). Cells were incubated for 20 minutes at room temperature (RT) in the dark, washed, fixed and resuspended in 200µl of phosphate-buffered saline (PBS) for acquisition on the BD LSRFortessa™ X-20 (BD Biosciences).

### Stimulation of PBMCs using TLR ligands and intracellular cytokine staining

To assess the functional capacity of monocytes, mDCs, and pDCs, PBMCs (1x10^6^ cells/ml) were stimulated with a range of toll-like receptor (TLR) ligands; lipopolysaccharides (LPS) for TLR4, CL097 for TLR7/8 and oligodeoxynucleotides containing unmethylated CG dinucleotides (CpG-ODN22) for TLR9 as previously described (34). PBMCs were stimulated with TLR ligands according to the following conditions: a control (unstimulated) i.e. PBMCs reconstituted in R10 (RPMI + 10% FBS+ 1% pen strep) medium only and PBMCs incubated separately with the following TLR ligands (TLR4-LPS, TLR7/8-CL097 and TLR9-CpG-ODN22) reconstituted in R10. The cells were incubated for 18 hours at 37°C CO_2_ in the presence of brefeldin A (5µg/ml) (Sigma-Aldrich, St. Louis). The cells were washed, and stained with a cocktail of antibodies: CD56-BV510 (1H11), CD19-BV510 (HIB19), CD3-BV650 (OKT3), CD4-APC (OKT4), CD14-APC-CY7 (63D3), CD11c-PE-CY5 (3.9), CD123-BV42 (6H6) all from (BioLegend, San Jose, USA), HLA-DR-PECF594 (L243), CD8-FITC (HIT8a) (BD Biosciences, San Jose, USA), washed then intracellularly stained with a cocktail of antibodies: interferon-alpha (IFN-α-PE) (7N4-1) (BD Biosciences, San Jose, USA), tumour necrosis factor-alpha (TNF-α-BV605) (MAb11) and interleukin 1-beta (IL-1β-APC) (REA1172) (BioLegend, San Jose, USA) in the presence of permeabilising solution PERM B (Invitrogen, Carlsbad, California, USA). Cells were incubated for 20 minutes at RT in the dark, washed, fixed and resuspended in 200µl of PBS for acquisition on the BD LSRFortessa™ X-20 (BD Biosciences). Data was acquired using the FACSDiva software (BD, Biosciences, San Jose, USA). Each flow cytometry run recorded a total range of 500,000–1x10^6^ events. The acquired data was further analyzed using FlowJo software (TreeStar, Inc., Ashland, Oregon, USA).

### Assessment of plasma biomarkers

Monocyte activation and coagulation activity were assessed in the plasma of the study participants by measuring the expression levels of human soluble (sCD14) using DuoSet ELISA and D-dimer using commercially available enzyme-linked immunosorbent assay (ELISA) kits from (R&D Systems, Minnesota, USA).

### Data acquisition

Statistical analyses were performed using GraphPad Prism version 8.01 (GraphPad Software, La Jolla, California, USA). CD4+ and CD8+ T cell (HLA-DR and CD38 expression) activation and monocyte subset frequencies between the different groups were analysed using One-way ANOVA and unpaired t-test for multiple and single-group comparisons in normally distributed data. Cytokine (TNF-α, IFN-α and IL-1β) production in APCs after TLR ligand stimulation, plasma biomarkers (sCD14 and D-dimer) and CD69 and CD86 expression on APCs and T cells between the different groups were analyzed using Kruskal-Wallis test for multiple group comparisons and Mann-Whitney *U* test for single group comparisons in non-parametric data. Spearman’s rank correlation coefficient was used to analyse the relationship between activation markers on T cells and monocytes. Differences were considered statistically significant at *P* < 0.05.

## Results

### Clinical characteristics of study participants

The study included 73 participants divided into four groups: people living with HIV maintaining spontaneous viral control PWH_EC_ (n=16), HIV progressors PWH_PROG_ (n=20), people living with HIV suppressed on ART PWH_ART_ (n=19) and a control group without HIV PWOH_HIV-_ (n=18) (**Table 1**). The CD4+ T cell counts and the age range differed significantly across the groups (**Table 1**, **Supplementary Figures 1A, B**). We found that PWOH_HIV-_ were significantly younger than PWH_EC_ (p=0.01) and PWH_ART_ (p<0.0001). Additionally, PWH_ART_ were significantly older than PWH_EC_ (p=0.004), PWH_PROG_ (p<0.0001), and PWOH_HIV-_ (p<0.0001, **Supplementary Figure 1A**). CD4+ T cell count varied across the different groups; treatment naïve PWH_PROG_ had significantly lower CD4+ T cell count compared to PWH_EC_ (p=0.005) and PWH_ART_ (p=0.003, **Supplementary Figure 1B**). Furthermore, treatment naïve PWH_PROG_ had a significantly lower CD4+/CD8+ ratio compared to PWH_EC_ (p<0.0002) and PWH_ART_ (p=0.01, **Supplementary Figure 1C**). Overall, HIV-1 infection leads to a reduction in CD4+ T cell counts in treatment naïve PWH, including PWH_EC_. Additionally, the lower CD4+/CD8+ ratio observed in treatment naïve PWH_PROG_ suggests a higher risk of disease progression in this group.

**Table 1:** Clinical characteristics of study participants.

Groups	Number of participants	Age: median (IQR)	M/F	Viral load, RNA copies/ml: median (IQR)	CD4+ T cell count (cells/mm3): median (IQR)
PWOH_HIV-_	18	33 (29 – 37)	10/8	N/A	940 (785 – 1136)
PWH_EC_	16	40 (34 – 44)	3/13	<20	789 (582 – 1017)
PWH_ART_	19	47 (42 – 53)	7/12	<20	766 (605 – 814)
PWH_PROG_	20	35 (29 – 41)	6/14	38485 (8966 – 397088)	159 (74 – 556)

Keywords: people living with HIV who maintained spontaneous viral control (PWH_EC_), people living with HIV on ART and virally suppressed (PWH_ART_), people living with progressive HIV infection (PWH_PROG_), a control group of people without HIV (PWOH_HIV-_), N/A, not applicable; HIV, human immunodeficiency virus; ART, antiretroviral therapy.

### Elevated CD4+ and CD8+ T cell activation in treatment naïve PWH including PWH_EC_

**Figure 1:**
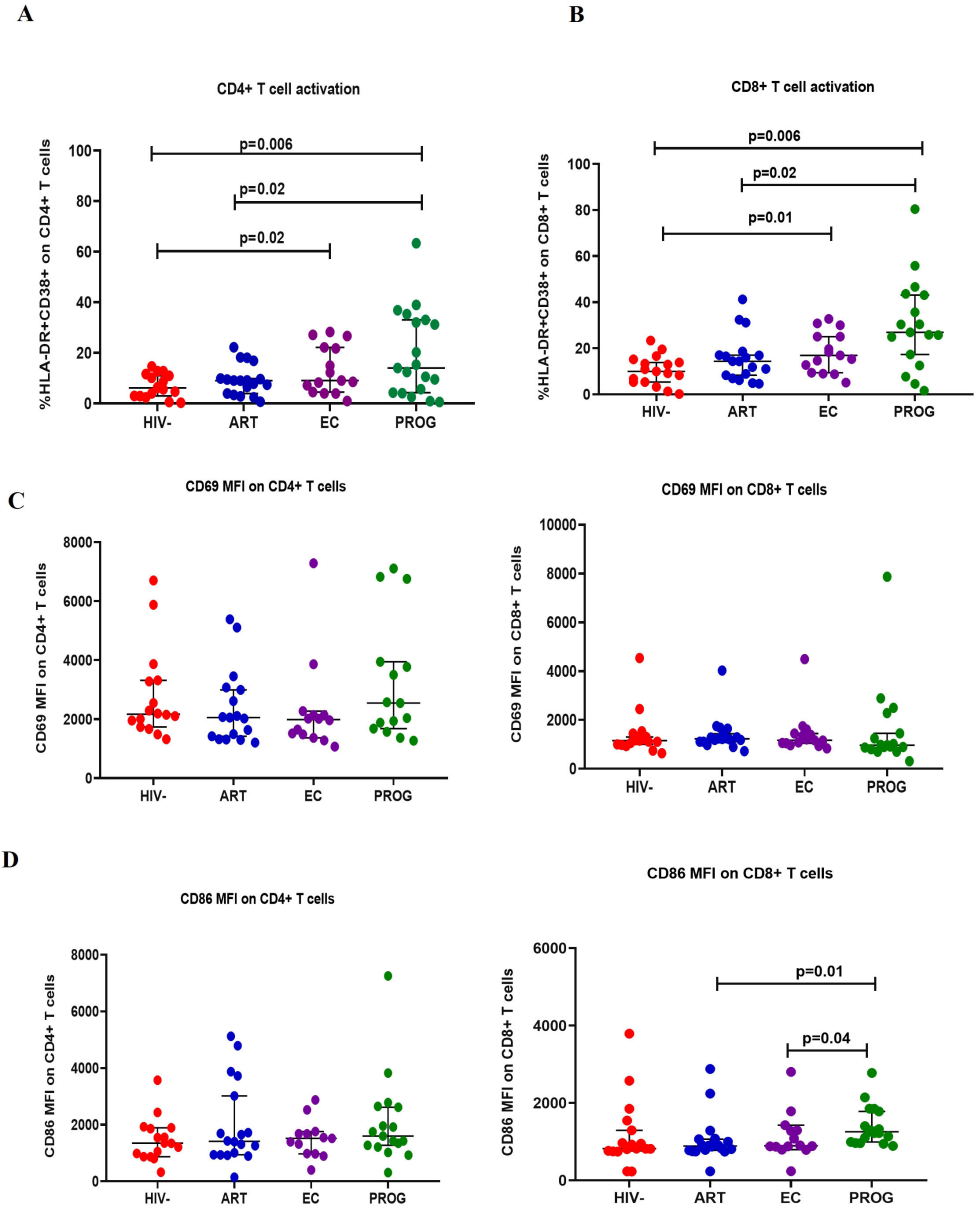
T cell activation. **(A)** CD4+ T cell and **(B)** CD8+ T cell activation were measured by co-expression of HLA-DR+ and CD38+ markers. **(C)** CD69 expression on CD4+ and CD8+ T cells. **(D)** CD86 expression on CD4+ and CD8+ T cells was assessed in people living with HIV who maintained spontaneous viral control PWH_EC_ (n=15), people living with HIV on ART for a minimum of 7 years and virally suppressed PWH_ART_ (n=18), people living with progressive HIV infection PWH_PROG_ (n=19), a group of control people without HIV PWOH_HIV-_ (n=17). Each dot represents an individual, and horizontal lines represent the medians with interquartile ranges. One-way ANOVA was used for normally distributed data and the Kruskal-Wallis test was for non-parametric data. The Mann-Whitney *U* test was used to assess differences between the respective groups. Data is expressed as the % frequency of the total parent cells for T cell activation and mean fluorescence intensity (MFI) for CD69 and CD86 expression. Significant P values are shown (P<0.05). Four study participants (one from each group) were excluded due to insufficient PBMC yields and fewer cells acquired during sample acquisition. The x-axis displays patient groups.

We evaluated CD4+ and CD8+ T cell activation by assessing the co-expression of HLA-DR and CD38 positive cells expressed as % frequency, and early activation marker CD69 and co-stimulatory marker CD86 expressed as median fluorescence intensity (MFI) across the study groups. The gating strategy is shown in **Supplementary Figure 2**. Significantly higher co-expression of HLA-DR and CD38 on CD4+ and CD8+ T cells were observed in PWH_PROG_ and PWH_EC_ than in PWOH_HIV-_. PWH_ART_ had lower CD4+ and CD8+ T cell activation levels than treatment naïve PWH_PROG_ (all p<0.05, **Figures 1A, B**). CD69 is an activation marker and CD86 is a co-stimulatory marker expressed in the initial phases of T cell activation (31, 36). We assessed CD69 and CD86 on T cells. There were no significant differences in CD69 expression on T cells (**Figure 1C**) or in CD86 expression on CD4+ T cells between study groups, except for a lower expression of CD86 in CD8+ T cells of PWH_ART_ (p=0.01) and PWH_EC_ (p=0.04), compared to PWH_PROG_ (**Figure 1D**). Overall, this data shows significantly elevated CD4+ and CD8+ T cell activation (co-expression HLA-DR and CD38) in ART naïve PWH, including PWH_EC,_ compared to PWOH_HIV-_.

### Altered monocyte subset frequencies across all PWH groups, including PWH_EC_

**Figure 2:**
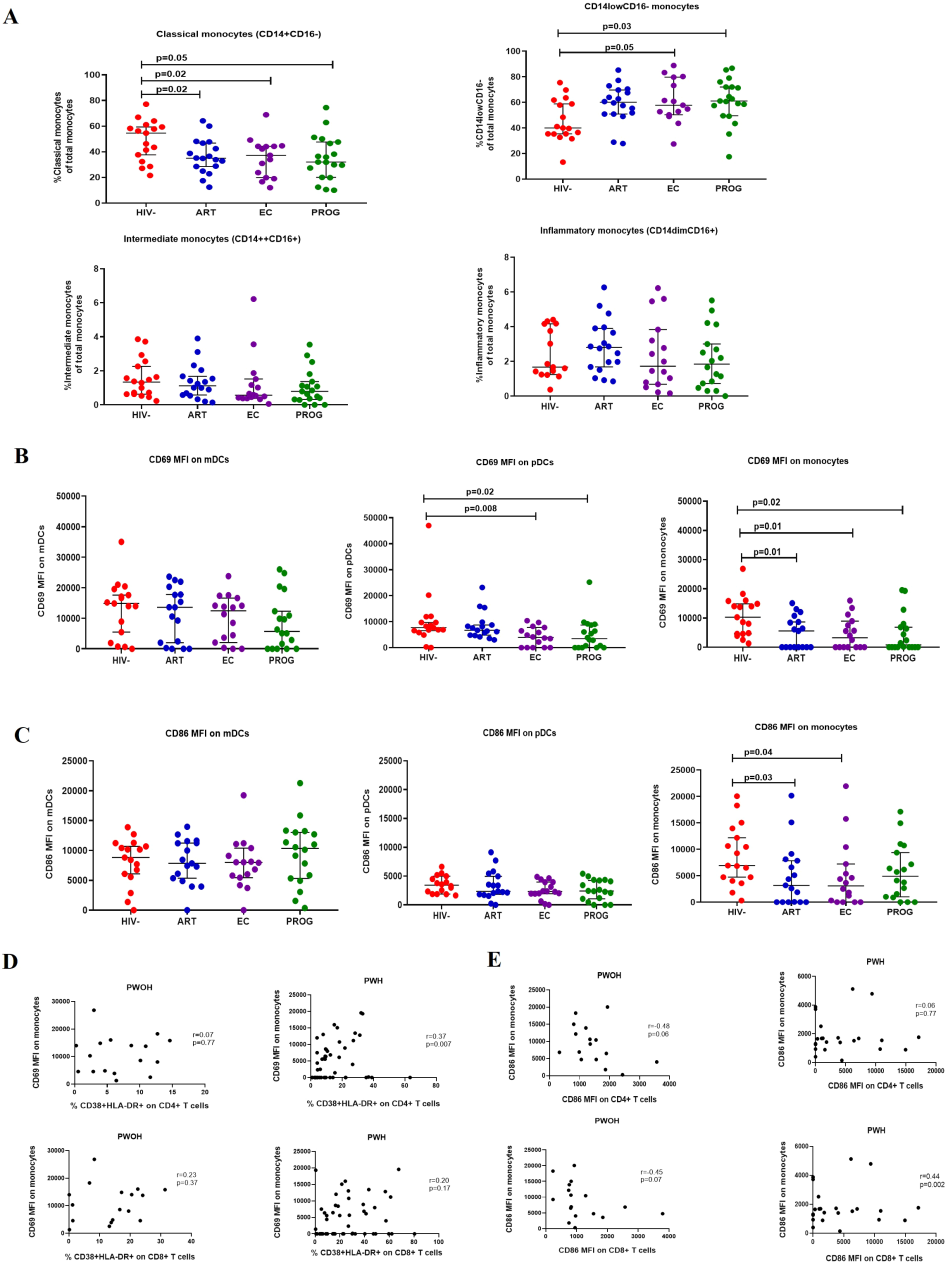
Frequencies of monocyte subsets. **(A)** classical, intermediate, inflammatory, and CD14lowCD16- monocytes. **(B)** CD69 expression and **(C)** CD86 expression on mDCs, pDCs, and monocytes. **(D)** Correlation between CD69 expression on monocytes and T cell activation. **(E)** Correlation between CD86 expression on monocytes and T cells of PWH_EC_ (n=15), PWH_ART_ (n=17), PWH_PROG_ (n=18_)_, (all grouped as PWH) and PWOH_HIV-_ (n=17). Each dot represents an individual, and horizontal lines represent the median with the interquartile range. One-way ANOVA was used to assess the differences between normally distributed data. The Kruskal-Wallis test was used to assess the differences in non-parametric data. An unpaired t-test (Mann-Whitney *U* test) was used to assess the differences between the respective groups. Monocyte subset frequencies are expressed as the % frequency of the total parent cells. Fluorescence minus one (FMO) control for CD69 and CD86 are shown. CD69 and CD86 data are expressed as median intensity frequency (MFI). p-values and Spearman rho (r) values are shown for correlation. *P<0.05* was considered statistically significant. Six study participants were excluded due to low PBMCs numbers and fewer cells acquired during sample acquisition. The x-axis displays patient groups.

Next, we characterised innate immune profiles by assessing the frequency of monocyte subtypes *i.e.* classical (CD14++CD16-), intermediate (CD14++CD16+), inflammatory (CD14+CD16+), CD14lowCD16- and dendritic cell subsets mDCs (CD11c+CD123-) and pDCs (CD11c-CD123+) in the study groups. The gating strategy is shown in **Supplementary Figure 3**. Frequencies of classical monocytes were significantly reduced in PWH_PROG_ (p=0.05), PWH_EC_ (p=0.02), and PWH_ART_ (p=0.02) in comparison to PWOH_HIV-._ In contrast, the levels of CD14lowCD16- subset were significantly elevated in PWH_PROG_ (p=0.03) and PWH_EC_ (p=0.05) compared to PWOH_HIV-._ (**Figure 2A**). There was no significant difference in the frequencies of inflammatory and intermediate monocytes between the study groups (**Figure 2A**). Our results suggest that HIV-1 impacts the frequencies of the classical monocytes, which was observed across all groups irrespective of viremia and treatment status. However, CD14lowCD16- monocytes were not significantly modified in the PWH_ART_ group, suggesting partial restoration or preservation of this subset with antiretroviral therapy.

### Altered expression of activation markers CD69 and CD86 on monocytes and pDCs across all PWH groups, including PWH_EC_

CD69 is an activation marker expressed in the initial phases of T cell activation and a critical marker of activation and functional state of innate immune cells (31, 36). We assessed the activation of innate immune cells (monocytes and dendritic cells) by measuring the expression (MFI) of the early activation marker, CD69, and the co-stimulatory marker, CD86 across all study groups. No significant differences were observed in CD69 expression on mDCs between the respective groups. However, CD69 expression on pDCs was significantly lower in PWH_EC_ (p=0.008) and PWH_PROG_ (p=0.02) compared to PWOH_HIV-._ Similarly, CD69 expression on monocytes was significantly lower in PWH_PROG_ (p=0.02), PWH_EC_ (p=0.01) and PWH_ART_ (p=0.01) compared to PWOH_HIV-_ (**Figure 2B**). No significant differences in CD86 expression were observed in mDCs and pDCs between the respective groups, however, CD86 expression on monocytes was significantly lower in PWH_EC_ (p=0.04) and PWH_ART_ (p=0.03) compared to PWOH_HIV-_ (**Figure 2C**). These findings demonstrate a consistent reduction in activation markers CD69 and CD86 on monocytes and pDCs across all PWH groups including elite controllers, indicating persistent innate immune modulation despite viral control. We assessed the relationship between the CD69 and CD86 expression on innate immune cells (monocytes, mDCs and pDCs) and CD38 and HLA-DR co-expression on CD4+ and CD8+ T-cells across the respective groups. We found a significant positive correlation between CD69 expression on monocytes and activated CD4+ T cells of PWH (r=0.37, p=0.007, **Figure 2D**). Additionally, there was a positive correlation between CD86 expression on monocytes and activated CD8+ T cells in PWH (r=0.44, p=0.002, **Figure 2E**). These results suggest that in HIV-1 infection, increased monocyte activation is associated with elevated T cell activation.

### Reduced production of IL-1β and TNF-α by monocytes from PWH_EC_ and PWH_PROG_ compared to PWOH_HIV-_ post-stimulation with TLR4 and TLR7/8 ligands

**Figure 3:**
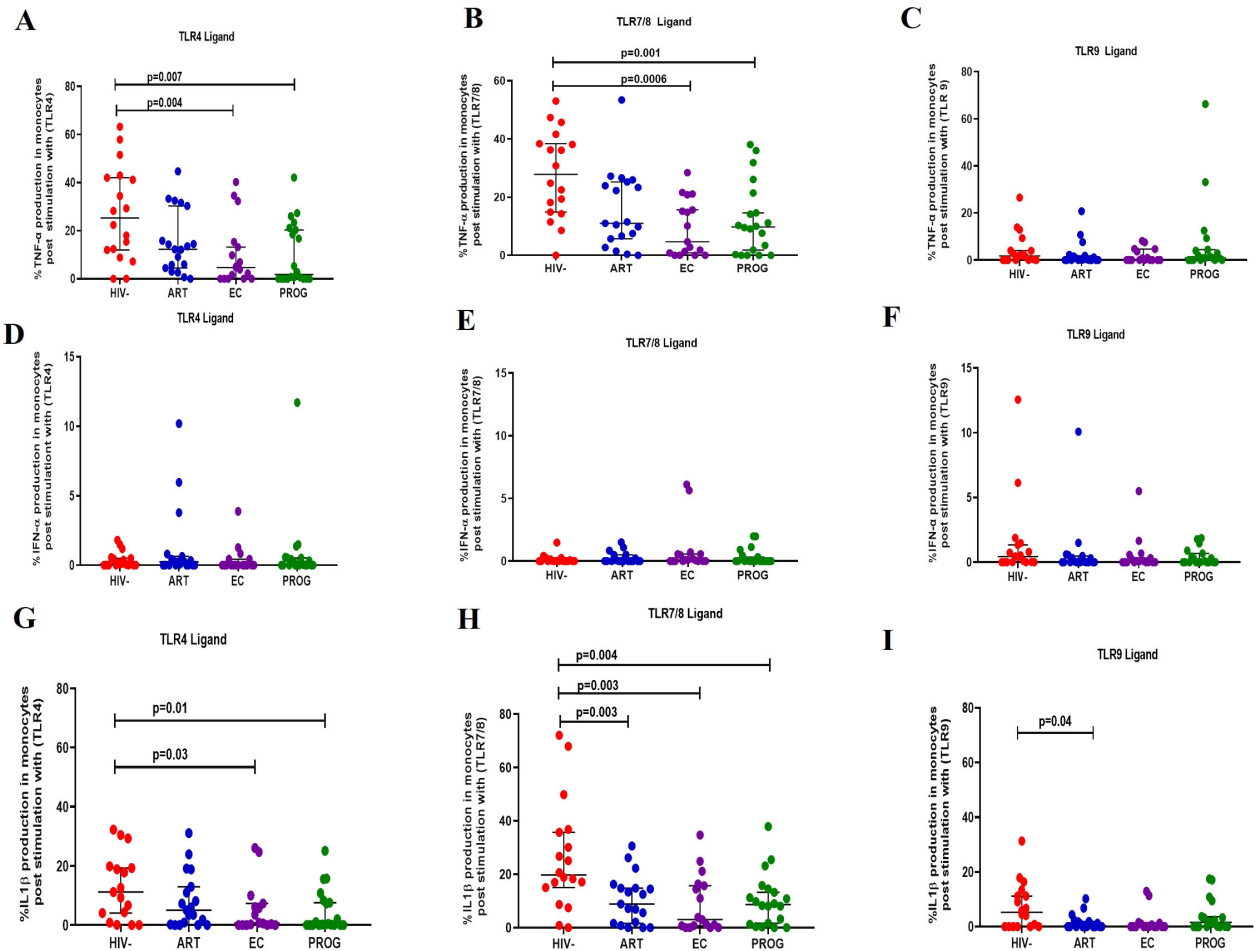
Monocyte production of cytokine in response to TLR ligand stimulation. Monocyte production of TNF-α, IFN-α and IL-1β was measured following stimulation with TLR4-LPS, TLR7/8-CL097 and TLR9-CpG-ODN22 in PWH_EC_ (n=16), PWH_ART_ (n=19), PWH_PROG_ (n=18_)_, and PWOH_HIV-_ (n=18). Panels **(A–C)** show TNF-α production after stimulation with ligand TLR4-LPS **(A)**, TLR7/8-CL097 **(B)**, and TLR9-CpG-ODN22 **(C)**. Panels **(D–F)** show IFN-α production after stimulation with ligand TLR4-LPS **(D)**, TLR7/8-CL097 **(E)**, and TLR9-CpG-ODN22 **(F)**. Panels **(G–I)** show IL-1β production after stimulation with ligand TLR4-LPS **(G)**, TLR7/8-CL097 **(H)**, and TLR9-CpG-ODN22 **(I)**. Each dot represents an individual, and horizontal lines represent the median with the interquartile range. The Kruskal-Wallis test was used to assess the differences in non-parametric data. An unpaired t-test (Mann-Whitney *U* test) was used to assess the differences between the respective groups. *P<0.05* was considered statistically significant. Two study participants from PWH_PROG_ were excluded due to low PBMCs numbers and fewer cells acquired during sample acquisition. The x-axis displays patient groups.

We assessed the functional capacity of monocytes by measuring their ability to secrete pro-inflammatory cytokines (TNF-α, IFN-α and IL-1β) after stimulation with TLR4 (LPS)/TLR7/8 (CL097) and TLR9 (CpG-ODN22) ligands in the respective groups. The representative gating strategy is shown in **Supplementary Figure 4**. There was a significant reduction in TNF-α production by monocytes from PWH_PROG_ and PWH_EC_ compared to PWOH_HIV-_ after stimulation with TLR4 and TLR7/8 ligands (all p<0.05) (**Figures 3A, B**), but not after stimulation with TLR9 ligand (**Figure 3C**). IFN-α production did not differ between the study groups for any of the stimuli evaluated (**Figures 3D–F**). IL-1β production was lower in monocytes from PWH_EC_ (p=0.03) and PWH_PROG_ (p=0.01) compared to PWOH_HIV-_ following TLR4 ligand stimulation (**Figure 3G**). After TLR7/8 ligand stimulation, monocytes from PWH_EC_ (p=0.003), PWH_PROG_ (p=0.004) and PWH_ART_ (p=0.003) produced significantly lower levels of IL-1β compared to PWOH_HIV-_ (**Figure 3H**). In addition, following TLR9 stimulation, monocytes from PWH_ART_ (p=0.04) produced significantly lower levels of IL-1β compared to PWOH_HIV-_ (**Figure 3I**). Overall, these results suggest that monocytes from PWH exhibit a reduced capacity to secrete TNF-α and IL-1β upon TLR stimulation, irrespective of treatment status.

### Reduced production of IL-1β and TNF-α in dendritic cells of PWH_EC_ and PWH_PROG_ compared to PWOH_HIV-_ after stimulation with TLR7/8 ligand

Next, we assessed the functional capacity of plasmacytoid dendritic cells (pDCs) and myeloid dendritic cells (mDCs) by evaluating their ability to secrete TNF-α, IFN-α and IL-1β after TLR4 (LPS), TLR7/8 (CL097) and TLR9 (CpG-ODN22) stimulation.

#### Plasmacytoid dendritic cells

**Figure 4:**
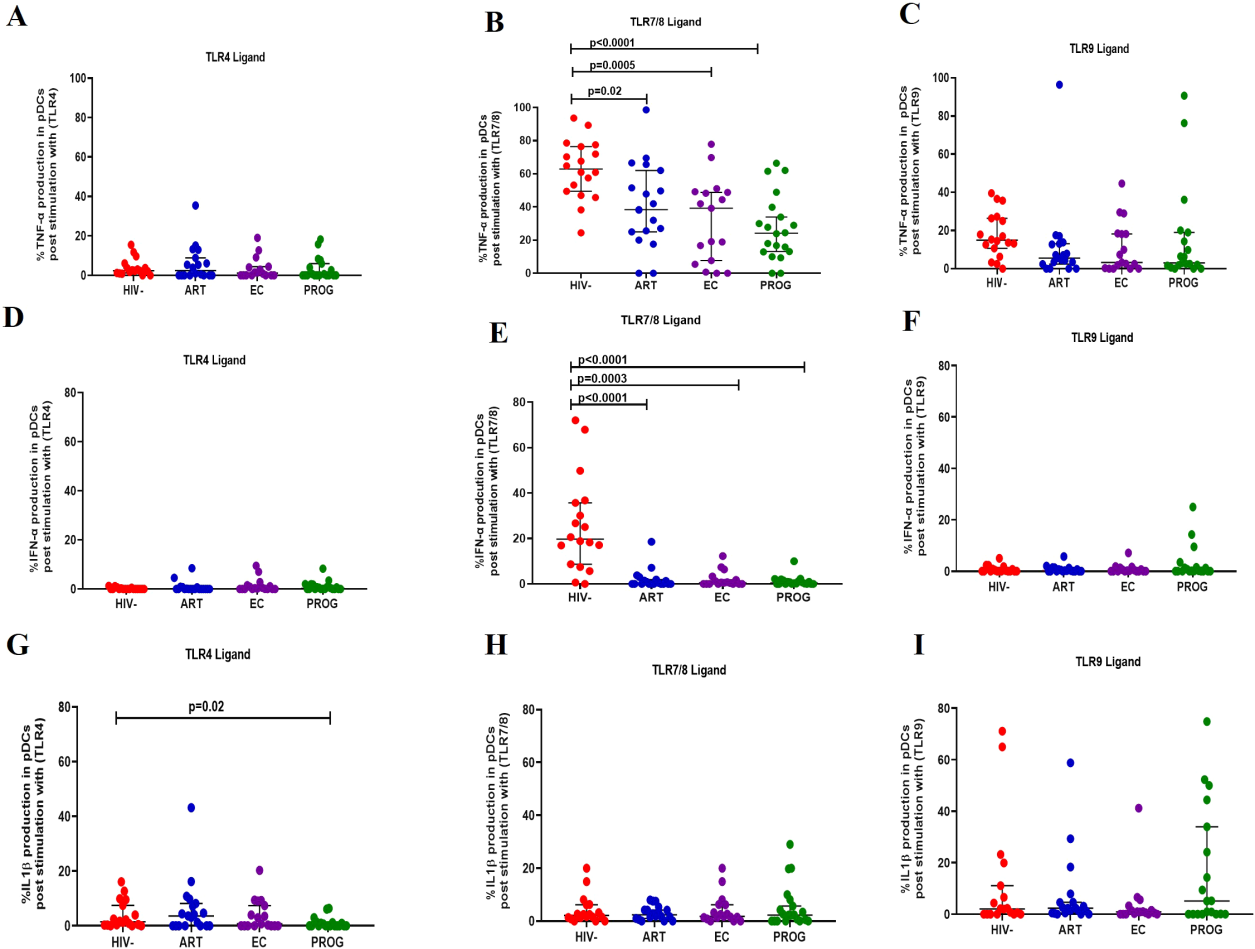
pDC production of cytokine in response to TLR ligand stimulation. pDC production of TNF-α, IFN-α and IL-1β was measured following stimulation with TLR4-LPS, TLR7/8-CL097 and TLR9-CpG-ODN22 in PWH_EC_ (n=16), PWH_ART_ (n=19), PWH_PROG_ (n=18_)_, and PWOH_HIV-_ (n=18). **(A–C)** show TNF-α production after stimulation with ligand TLR4-LPS **(A)**, TLR7/8-CL097 **(B)**, and TLR9-CpG-ODN22 **(C)**. **(D–F)** show IFN-α production after stimulation with ligand TLR4-LPS **(D)**, TLR7/8-CL097 **(E)**, and TLR9-CpG-ODN22 **(F)**. Panels **(G–I)** show IL-1β production after stimulation with ligand TLR4-LPS **(G)**, TLR7/8-CL097 **(H)**, and TLR9-CpG-ODN22 **(I)**. Each dot represents an individual, and horizontal lines represent the median with the interquartile range. One-way ANOVA was used to assess the differences between normally distributed data. The Kruskal-Wallis test was used to assess the differences in non-parametric data. An unpaired t-test (Mann-Whitney *U* test) was used to assess differences between the respective groups. *P<0.05* was considered statistically significant. Two study participants from PWH_PROG_ group were excluded from the analysis due to low PBMCs numbers and fewer cells acquired during sample acquisition. The x-axis displays patient groups.

pDCs stimulated with TLR4 and TLR9 ligand demonstrated no significant difference in the production of TNF-α across the groups (**Figures 4A, C**). After stimulation with TLR7/8 ligand, pDCs from PWH_EC_ (p=0.0005), PWH_PROG_ (p<0.0001) and PWH_ART_ (p=0.02) produced significantly lower levels of TNF-α compared to PWOH_HIV-_ (**Figure 4B**). Similarly, no significant differences were found in the production of IFN-α across the different groups after stimulation of pDCs with TLR4 and TLR9 ligand (**Figures 4D, F**). Following stimulation with TLR7/8 ligand, pDCs from PWH_EC_ (p=0.0003), PWH_PROG_ (p<0.0001) and PWH_ART_ (p<0.0001) produced significantly lower levels of IFN-α compared to PWOH_HIV-_ (**Figure 4E**). Finally, after stimulation with TLR4 ligand, pDCs from PWH_PROG_ (p=0.02) produced significantly lower levels of IL-1β compared to PWOH_HIV-_ (**Figure 4G**). No significant differences in IL-1β production by pDCs were observed across the different groups after stimulation with TLR7/8 and TLR9 ligands (**Figures 4H–I**). Taken together, these results show that pDCs from PWH display a reduced capacity to produce TNF-α and IFN-α following TLR7/8 stimulation.

#### Myeloid dendritic cells

**Figure 5:**
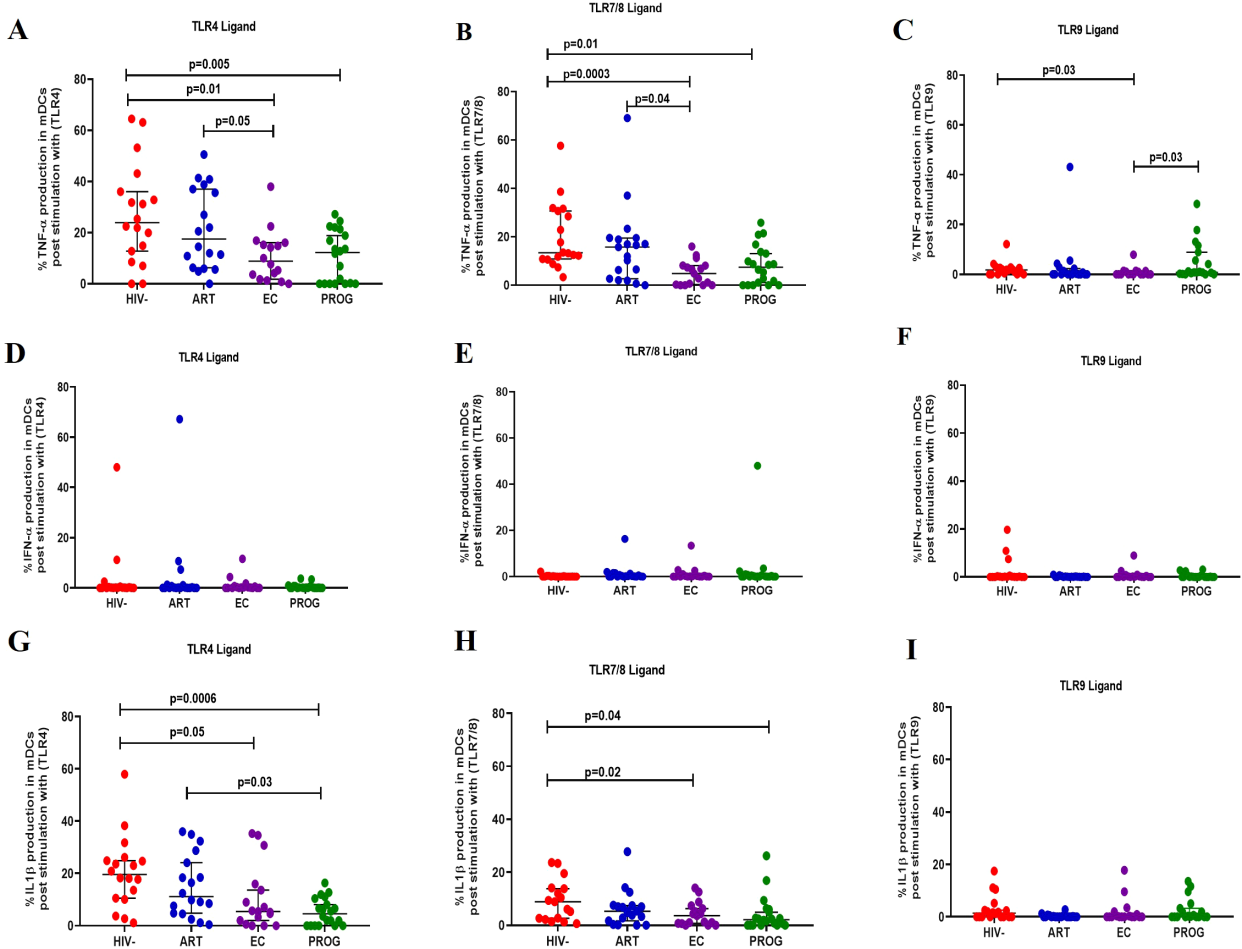
mDC cytokine production in response to different TLR ligand stimulation. mDC production of TNF-α, IFN-α and IL-1β was measured following stimulation with TLR4-LPS, TLR7/8-CL097 and TLR9-CpG-ODN22 in PWH_EC_ (n=16), PWH_ART_ (n=19), PWH_PROG_ (n=18_)_, and PWOH_HIV-_ (n=18). **(A–C)** show TNF-α production after stimulation with ligand TLR4-LPS **(A)**, TLR7/8-CL097 **(B)**, and TLR9-CpG-ODN22 **(C)**. **(D–F)** show IFN-α production after stimulation with ligand TLR4-LPS **(D)**, TLR7/8-CL097 **(E)**, and TLR9-CpG-ODN22 **(F)**. **(G–I)** show IL-1β production after stimulation with ligand TLR4-LPS **(G)**, TLR7/8-CL097 **(H)**, and TLR9-CpG-ODN22 **(I)**. Each dot represents an individual, and horizontal lines represent the median with the interquartile range. The Kruskal-Wallis test was used to assess the differences in non-parametric data. Unpaired t-tests (Mann-Whitney *U* test) were used to assess differences between the respective groups. *P<0.05* was considered statistically significant. Two study participants from PWH_PROG_ were excluded from the analysis due to low PBMCs numbers and fewer cells acquired during sample acquisition. The x-axis displays patient groups.

Following stimulation with TLR4 ligand, mDCs from PWH_PROG_ (p=0.005) and PWH_EC_ (p=0.01) produced significantly lower levels of TNF-α compared to PWOH_HIV-_, and lesser levels in treatment naïve PWH_EC_ (p=0.05) compared to PWH_ART_ after stimulation with TLR4 ligand (**Figure 5A**). Additionally, reduced levels of TNF-α were observed in PWH_EC_ (p=0.0003) and PWH_PROG_ (p=0.01) compared to PWOH_HIV-_ after stimulation with TLR7/8 ligand. In contrast, PWH_ART_ had a significantly higher functional capacity than PWH_EC_ (p=0.04, **Figure 5B**). Interestingly, increased levels of TNF-α were noted in PWH_PROG_ compared to PWH_EC_ (p=0.03) after stimulation with TLR9 ligand. Furthermore, reduced levels of TNF-α were observed in PWH_EC_ (p=0.03) compared to PWOH_HIV-_ after stimulation with TLR9 ligand (**Figure 5C**). No significant differences were observed in the production of IFN-α in mDCs between the different groups (**Figures 5D–F**). IL-1β production was lower in treatment naïve PWH_PROG_ compared to PWH_ART_ (p=0.03), this reduction was also observed in PWH_EC_ (p=0.05) and PWH_PROG_ (p=0.006) compared to PWOH_HIV-_ after stimulation with TLR4 ligand (**Figure 5G**). Lower production of IL-1β was observed in mDCs of PWH_EC_ (p=0.02) and PWH_PROG_ (p=0.04) compared to PWOH_HIV-_ after stimulation with TLR7/8 ligand (**Figure 5H**). No significant differences were observed in the production of IL-1β-α in mDCs between the different groups after stimulation with TLR9 ligand (**Figure 5I**). In conclusion, IL-1β production decreased in all the PWH groups, likely due to HIV-1 infection. However, the ART group showed elevated IL-1β levels, suggesting that treatment could restore the functional capacity of mDCs.

### Elevated levels of D-dimer and sCD14 in treatment naïve PWH compared to PWOH_HIV-_

**Figure 6:**
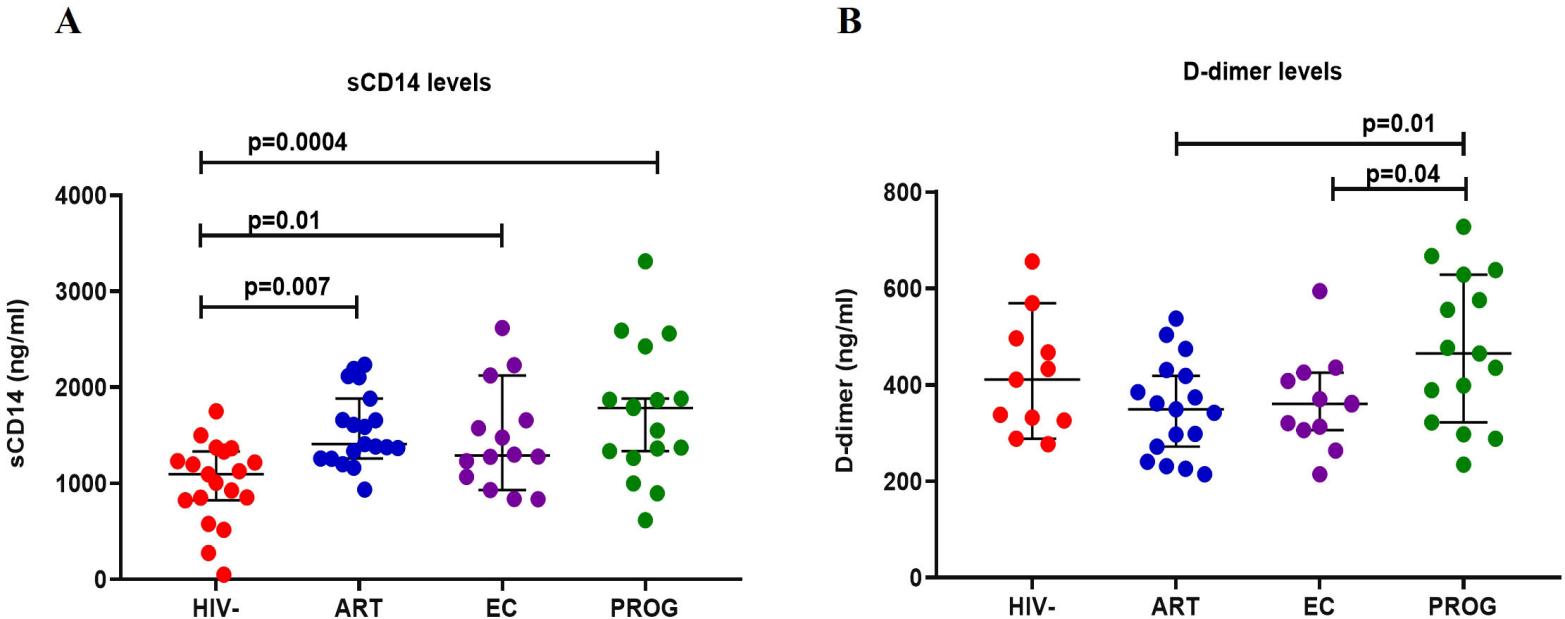
Measurement of plasma biomarkers levels in study participants. **(A)** D-dimer and **(B)** sCD14 in PWH_EC_ (n=14), PWH_ART_ (n=19), PWH_PROG_ (n=17_)_, and PWOH_HIV-_ (n=19). Each dot represents an individual, and horizontal lines represent the median with the interquartile range. One-way ANOVA was used to assess the differences between normally distributed data. The Kruskal-Wallis test was used to assess the differences in non-parametric data. An unpaired t-test (Mann-Whitney *U* test) was used to assess differences between the respective groups. *P<0.05* was considered statistically significant. For sCD14, five participants and for D-dimer, 18 participants were excluded from the analysis because their concentration levels were outside the range of the standard curve. The x-axis displays patient groups.

Elevated levels of sCD14 and D-dimer have been reported in PWH and are indicators of chronic inflammation and immune activation – factors which contribute to non-AIDS-related comorbidities (37). Thus, we analysed plasma levels of sCD14, a marker of monocyte activation, and D-dimer, a marker of non-AIDS-related cardiovascular events (37). sCD14 levels were significantly increased in PWH_EC_ (p=0.01), PWH_ART_ (p=0.007) and PWH_PROG_ (p=0.0004) compared to PWOH_HIV-_ (**Figure 6A**). Furthermore, as expected, PWH_PROG_ had significantly elevated D-dimer levels compared to PWH_ART_ (p=0.01) and PWH_EC_ (p=0.04) (**Figure 6B**). Overall, these results demonstrate increased monocyte activation (sCD14) in PWH, including PWH_EC_, compared to PWOH_HIV-._ This indicates elevated monocyte activation despite ART treatment and low viremia. Treatment naïve PWH_PROG_ individuals exhibited higher D-dimer levels, suggesting a greater risk of developing cardiovascular events compared to PWH_ART_ and PWH_EC_.

## Discussion

Elite controllers are an ideal model for an HIV-1 functional cure due to their ability to suppress viral replication and maintain a relatively functional immune system without any drug therapy (12, 38). Studies have highlighted both viral and host cell factors as the basis for spontaneous viral control. Specifically, HIV-1 specific CD8+ T cell responses have been thoroughly investigated. However, approximately 70% of HIV controllers do not present with this adaptive immunity phenotype (11, 14, 39), emphasizing the need to explore the role and mechanisms of innate immune cells (monocytes, mDCs and pDCs) on spontaneous viral control. In this study, we examined the frequency, function and activation of monocytes, mDCs and pDCs across the different groups of PWH. Our findings show elevated T cell activation in treatment naïve PWH groups; specifically, PWH_EC_ and PWH_PROG_ had increased CD8+ T cell activation compared to PWH_ART_ and PWOH_HIV-_. Secondly, PWH_EC_ exhibited reduced expression of CD69 and CD86 in monocytes compared to PWOH_HIV-_. Meanwhile, PWH_EC_ and PWOH_ART_ displayed reduced expression of CD86 in CD8+T cells compared to PWH_PROG_. We also observed a significant decrease in the classical monocyte subset (CD14++CD16-) and an increase in CD14lowCD16- frequencies in all treatment naïve PWH compared to PWOH_HIV-_. Impaired inflammatory cytokines production (TNF-α, IFN-α and IL-1β) by monocytes and dendritic cells was noted in PWH_EC_, while treatment with ART in PWH_ART_ improved the ability of APCs to produce TNF-α and IL-1β. Finally, elevated plasma levels of sCD14 (monocyte activation) and D-dimer (cardiovascular health) were observed in PWH groups compared to PWOH_HIV-_.

Upon HIV-1 infection, immune cell activation increases to control viral replication and seek to eradicate the virus (40). In agreement with previous studies of elevated T cell activation in PWH (41–43), we found significantly higher levels of CD8+ T cell activation in treatment naïve PWH_PROG_ and PWH_EC_ compared to PWH_ART_ and PWOH_HIV-_. PWH_EC_ are reported to exhibit a distinct CD8+ T cell phenotype, including enhanced polyfunctionality, cytolytic activity, proliferative capacity and more differentiated memory CD8+ T cells, contributing to spontaneous viral control (44–47). Although CD8+ T cell function was not assessed in our study, we speculate that the observed high CD8+ T cell activation in PWH_EC_ may contribute to spontaneous viral control. Furthermore, a lower CD4/CD8 ratio was observed in the treatment naïve PWH_PROG_ compared to PWH_EC_ and PWH_ART,_ suggesting immune activation and an increased likelihood of developing non-AIDs-related comorbidities. A higher CD4/CD8 ratio is associated with improved health outcomes (48). Therefore, the preserved CD4 T cell levels in PWH_EC_ and PWH_ART_ in our study may account for the high CD4/CD8 ratio and more favourable disease outcomes.

We found a pattern of downmodulation of CD69 and CD86 on monocytes and CD8+ T cells from PWH_EC_ and PWH_PROG_ compared to PWOH_HIV-_. Interestingly, although ART improved CD86 expression, it had minimal effect on CD69 levels. Similar observations were made by Naidoo et al. showing reduced CD86 expression levels on dendritic cells and monocytes in a South African cohort of PWH in both the hyperacute and chronic phases of ART treatment (34). Several other studies did not observe an increase in CD86 levels on monocytes, including monocytes from patients with systemic lupus erythematosus (49–51). CD86 on APCs interacts with the CD28 receptor complex and cytotoxic T-lymphocyte associated protein 4 (CTLA-4) on T cells, providing co-stimulation for T cell activation and proliferation to enhance responses against pathogens (36). Chaudhry et al. demonstrated that HIV-1 Nef protein reduces the surface expression of CD86 in APCs, affecting naïve T-cell activation (52). Therefore, we postulate that the observed decrease in CD86 expression on monocytes from PWH_EC_ may indicate an HIV-1 induced impairment in monocyte antigen presentation capacity, like that observed in PWH_PROG,_ and ART does not seem to completely restore CD86 levels.

Monocytes play a crucial role in the immune response against HIV-1 by producing inflammatory cytokines (TNF-α and IL-1β), expressing co-stimulatory molecules, and presenting antigens to T cells (53). Our results demonstrate reduced classical monocyte (CD14++CD16-) frequencies in all PWH groups, including PWH_ART_ and PWH_EC_ compared to PWOH_HIV-_, consistent with previous reports from Asian cohorts of PWH and individuals with acute coronary syndrome (54–57). ART did not restore classical monocyte frequencies in PWH compared to those observed in PWOH_HIV-_. Naidoo et al. (34) found that very early ART (in the hyperacute phase) restored classical monocyte frequencies more effectively than later treatment initiation. Surprisingly, we found no significant differences in the frequencies of inflammatory or intermediate monocyte subsets between PWH and PWOH_HIV-_. This result is different to a previous study which reported elevated frequencies of inflammatory monocyte subset in PWH (58). These discrepancies may be due to several factors including small sample size, ethnicity and sex differences. A previous study has shown differences in immune responses between men and women (59). Our small sample size prevented sex matching. Furthermore, we observed an expansion in CD14lowCD16- monocyte subsets in PWH_EC_ and PWH_PROG_ compared to PWOH_HIV-_, consistent with findings from Naidoo et al. (34) in a South African PWH cohort. Although the specific role of this subset is less characterised compared to other monocyte subsets, increased frequencies of this subset are believed to play a role in immune activation, an impairment in T cell activation through the downregulation of CD86, lower CD4 T cell counts and higher viral loads (60). Taken together, our data suggest that South African PWH_EC_ have a similar monocyte subset phenotype as other groups of PWH, predisposing these individuals to ongoing immune activation, chronic inflammation and risk of the development of non-AIDS conditions.

Functional assessment of APCs demonstrated a reduced ability to secrete TNF-α, IFN-α and IL-1β after stimulation with TLR ligands in PWH. Specifically, PWH_EC_ and PWH_PROG_ displayed a pattern of significantly lower capacity to produce IL-1β and TNF-α in monocytes after stimulation with both TLR4 and TLR7/8 ligands compared to PWOH_HIV-_. Similar findings were reported in a cohort of South African PWH with HIV/TB co-infection (35). Furthermore, a recent study in an ART-treated South African cohort found a similar dysfunction in APC cytokine production (TNF-α and IFN-α), which was restored after 24 months of ART (34). Monocytes are the primary producers of TNF-α after stimulation with TLR4 ligand (lipopolysaccharide) (61). Therefore, the decline in TNF-α and IL-1β secretion observed in PWH_EC_ could indicate monocyte dysfunction associated with innate monocyte exhaustion in the chronic stage of inflammation (62, 63). We postulate that the dysfunction in monocytes and dendritic cells’ ability to secrete TNF-α and IL-1β may be due to underlying chronic inflammation. It is important to note that this study focused on measuring IL-1β production following TLR stimulation, rather than directly assessing inflammasome activation. While IL-1β secretion is often linked to inflammasome activity, monocytes have been shown to release IL-1β in response to TLR ligands alone, independent of a secondary activation signal (64–66).

sCD14 plasma levels were elevated in all PWH groups, including PWH_ART_ and PWH_EC_, compared to PWOH_HIV-_ suggesting increased monocyte activation, microbial translocation and an elevated risk of cardiovascular events (37, 67–69). Additionally, elevated D-dimer levels in treatment naïve PWH_PROG_ compared to PWH_EC_ suggest an increased risk of developing cardiovascular-related conditions in PWH_PROG_ (70). Crowell et al. (71) reported higher hospitalisation rates for cardiovascular-related conditions in PWH_EC_ compared to PWH_ART_. Although we did not monitor hospitalizations, our data suggest that PWH_EC_ may be susceptible to non-AIDS-related conditions due to elevated sCD14 and D-dimer plasma levels, highlighting the importance of monitoring cardiovascular health in these individuals.

This study has several limitations. PWH_EC_ and PWH_PROG_ are rare populations, resulting in a limited sample size; in many settings all newly diagnosed individuals are started on ART immediately, reducing the number of potential treatment naïve participants. Recruitment of PWH_EC_ is challenging because viral load assays are not routinely done at HIV diagnosis, and our definition requires prolonged follow-up to demonstrate durable HIV control. Additionally, we could not match participants by sex or age due to the small pool of eligible individuals, potentially skewing our results. Furthermore, the younger age of the HIV-negative group represents a limitation of the recruitment process, which was constrained by the demographic profile of volunteers at the NHLS Sandringham campus. As a result of the age and gender mismatch across the groups, immunological comparisons should be interpreted with caution. The use of cryopreserved PBMCs presented challenges regarding cell viability and yield, which may have impacted certain analyses. Additionally, it is possible that a recruited PWH_PROG_ might have been a PWH_EC_ in earlier years with subsequent loss of viral control, while the PWH_ART_ group were PWH_PROG_ before starting ART. It can be similarly argued that the latter might have a PWH_EC_ that lost viral control and initiated on ART. However, given how rare the elite control phenotype is, which includes those with a limited duration of viral control to those with exceptional elite control, this is unlikely to impact our present findings comparing these distinct groups of PWH. Additionally, we acknowledge that different TLR ligands vary in their capacity to induce specific cytokines depending on receptor expression across innate immune cell types. Due to the limited sample size, particularly in the elite controller group, we employed a streamlined stimulation protocol to ensure consistency across phenotypes. Notably, stimulation with CL097 (TLR7/8) yielded cytokine trends consistent with those observed using LPS and CpG-2216, reinforcing the robustness of our findings.

Our study revealed reduced innate immune activation, significant alterations in monocyte subset frequencies, and a reduced capacity of monocytes and dendritic cells to secrete TNF-α and IL-1β in both PWH_EC_ and PWH_PROG_ compared to PWOH_HIV-_, indicating innate immune dysfunction. While lower innate immune activation in PWH_EC_ may help maintain a balanced and effective immune response, preventing excessive inflammation and reducing inflammation, chronic immune activation in PWH_PROG_ leads to immune exhaustion and functional impairment (3, 12, 72, 73). Moreover, PWH_EC_’s soluble marker profiles show persistent immune activation, evidenced by elevated sCD14 levels. This persistent activation underscores the importance of continued monitoring and potential therapeutic interventions to reduce chronic inflammation. Overall, our findings deepen the understanding of complex immune dynamics in PWH, with particular emphasis on the unique immune profiles of people who are elite controllers (PWH_EC_). This population offers a valuable model for studying spontaneous viral control, and our data provide important insights into the innate immune mechanisms that may underlie this phenotype. Specifically, the observed cytokine production patterns and monocyte activation profiles in PWH_EC_ suggest that regulated innate immune responses, rather than hyperactivation, may play a protective role. These findings highlight the critical interplay between innate and adaptive immunity and underscore the relevance of PWH_EC_ in informing the design of more effective HIV treatments. By characterizing immune regulation in this distinct group, our study contributes to the broader understanding of HIV pathogenesis and supports the development of immunotherapeutic strategies aimed at mimicking elite control in the general HIV positive population.

## Data Availability

The original contributions presented in the study are included in the article/**Supplementary Material**. Further inquiries can be directed to the corresponding author.

## References

[1] UNAIDS. HIV and AIDS Estimates [Fact Sheet]. Geneva: Joint United Nations Programme on HIV/AIDS (UNAIDS) (2024).

[2] Ahmed D Roy D Cassol E. Examining relationships between metabolism and persistent inflammation in HIV patients on antiretroviral therapy. Mediators Inflammation. (2018) 2018:6238978. doi: 10.1155/2018/6238978, PMID: 30363715 PMC6181007

[3] Huber A Baas FS van der Ven AJ Dos Santos JC. Innate immune cell functions contribute to spontaneous HIV control. Curr HIV/AIDS Rep. (2025) 22:6. doi: 10.1007/s11904-024-00713-0, PMID: 39614998 PMC11608392

[4] Deeks SG Archin N Cannon P Collins S Jones RB de Jong MA. Research priorities for an HIV cure: International AIDS Society Global Scientific Strategy 2021. Nat Med. (2021) 27:2085–98. doi: 10.1038/s41591-021-01590-5, PMID: 34848888

[5] Pacheco YM Jarrín I Rosado I Campins AA Berenguer J Iribarren JA. Increased risk of non-AIDS-related events in HIV subjects with persistent low CD4 counts despite cART in the CoRIS cohort. Antiviral Res. (2015) 117:69–74. doi: 10.1016/j.antiviral.2015.03.002, PMID: 25766861

[6] Collins DR Gaiha GD Walker BD. CD8+ T cells in HIV control, cure and prevention. Nat Rev Immunol. (2020) 20:471–82. doi: 10.1038/s41577-020-0274-9, PMID: 32051540 PMC7222980

[7] Kammers K Chen A Monaco DR Hudelson SE Grant McAuley W Moore RD. HIV antibody profiles in HIV controllers and persons with treatment-induced viral suppression. Front Immunol. (2021) 12:740395. doi: 10.3389/fimmu.2021.740395, PMID: 34512672 PMC8428532

[8] Cohen MS Chen YQ McCauley M Gamble T Hosseinipour MC Kumarasamy N. Prevention of HIV-1 infection with early antiretroviral therapy. New Engl J Med. (2011) 365:493–505. doi: 10.1056/NEJMoa1105243, PMID: 21767103 PMC3200068

[9] Casado C Galvez C Pernas M Tarancon Diez L Rodriguez C Sanchez Merino V. Permanent control of HIV-1 pathogenesis in exceptional elite controllers: a model of sp*ontaneous cure* . Sci Rep. (2020) 10:1902. doi: 10.1038/s41598-020-58696-y, PMID: 32024974 PMC7002478

[10] Gonzalo-Gil E Ikediobi U Sutton RE. Focus: Infectious diseases: Mechanisms of virologic control and clinical characteristics of HIV+ elite/viremic controllers. Yale J Biol Med. (2017) 90(2):245–59., PMID: 28656011 PMC5482301

[11] Hartana CA Xu GY. Immunological effector mechanisms in HIV-1 elite controllers. Curr Opin HIV AIDS. (2021) 16:243–8. doi: 10.1097/COH.0000000000000693, PMID: 34270465 PMC8373669

[12] Shi Y Su J Chen R Wei W Yuan Z Chen X. The role of innate immunity in natural elite controllers of HIV-1 infection. Front Immunol. (2022) 13:780922. doi: 10.3389/fimmu.2022.780922, PMID: 35211115 PMC8861487

[13] Claireaux M Robinot R Kervevan J Patgaonkar M Staropoli I Brelot A. Low CCR5 expression protects HIV-specific CD4+ T cells of elite controllers from viral entry. Nat Commun. (2022) 13:521. doi: 10.1038/s41467-022-28130-0, PMID: 35082297 PMC8792008

[14] International Controllers Study HIV Pereyra F Jia X McLaren JP Telenti A de Bakker PIW. The major genetic determinants of HIV-1 control affect HLA class I peptide presentation. Science. (2010) 330:1551–7. doi: 10.1126/science.1195271, PMID: 21051598 PMC3235490

[15] Gebara NY El Kamari V Rizk N. HIV-1 elite controllers: an immunovirological review and clinical perspectives. J Virus Eradication. (2019) p:163–3. doi: 10.1016/S2055-6640(20)30046-7, PMID: 31700663 PMC6816117

[16] Arts RJ Moorlag SJ Novakovic B Li Y Wang SY Oosting M. BCG vaccination protects against experimental viral infection in humans through the induction of cytokines associated with trained immunity. Cell Host Microbe. (2018) 23:89–100.e5. doi: 10.1016/j.chom.2017.12.010, PMID: 29324233

[17] Netea MG Domínguez Andrés J Barreiro LB Chavakis T Divangahi M Fuchs E. Defining trained immunity and its role in health and disease. Nat Rev Immunol. (2020) 20:375–88. doi: 10.1038/s41577-020-0285-6, PMID: 32132681 PMC7186935

[18] Stienstra R Netea Maier RT Riksen NP Joosten LA Netea MG. Specific and complex reprogramming of cellular metabolism in myeloid cells during innate immune responses. Cell Metab. (2017) 26:142–56. doi: 10.1016/j.cmet.2017.06.001, PMID: 28683282

[19] Batohi N Shalekoff S Martinson NA Ebrahim O Tiemessen CT Thobakgale CF. HIV-1 elite controllers are characterized by elevated levels of CD69-expressing natural killer cells. J AIDS (JAIDS) (2024) 97:522–32. doi: 10.1097/QAI.0000000000003518, PMID: 39219024 PMC11540281

[20] Marras F Nicco E Bozzano F Di Biagio A Dentone C Pontali E. Natural killer cells in HIV controller patients express an activated effector phenotype and do not up-regulate NKp44 on IL-2 stimulation. Proc Natl Acad Sci. (2013) 110:11970–5. doi: 10.1073/pnas.1302090110, PMID: 23818644 PMC3718138

[21] Martin-Gayo E Yu XG. Dendritic cell immune responses in HIV-1 controllers. Curr HIV/AIDS Rep. (2017) 14:1–7. doi: 10.1007/s11904-017-0345-0, PMID: 28110421 PMC5552363

[22] Soumelis V Scott I Gheyas F Bouhour D Cozon G Cotte L. Depletion of circulating natural type 1 interferon-producing cells in HIV-infected AIDS patients. J Am Soc Haematology. (2001) 98:906–12. doi: 10.1182/blood.V98.4.906, PMID: 11493432

[23] Martín-Moreno A Muñoz-Fernández MA. Dendritic cells, the double agent in the war against hiv-1. Front Immunol. (2019) 10:2485. doi: 10.3389/fimmu.2019.02485, PMID: 31708924 PMC6820366

[24] Mitchell BI Laws EI Chow DC SahBandar IN Gangcuangco LM Shikuma CM. Increased monocyte inflammatory responses to oxidized LDL are associated with insulin resistance in HIV-infected individuals on suppressive antiretroviral therapy. Viruses. (2020) 12:1129. doi: 10.3390/v12101129, PMID: 33028018 PMC7601436

[25] Austermann J Roth J Barczyk-Kahlert K. The good and the bad: Monocytes’ and macrophages’ diverse functions in inflammation. Cells. (2022) 11:1979. doi: 10.3390/cells11121979, PMID: 35741108 PMC9222172

[26] Marshall JS Warrington R Watson W Kim HL. An introduction to immunology and immunopathology. Allergy Asthma Clin Immunol. (2018) 14:49. doi: 10.1186/s13223-018-0278-1, PMID: 30263032 PMC6156898

[27] Sampath P Moideen K Ranganathan UD Bethunaickan R. Monocyte subsets: phenotypes and function in tuberculosis infection. Front Immunol. (2018) 9:1726. doi: 10.3389/fimmu.2018.01726, PMID: 30105020 PMC6077267

[28] Krishnan S Wilson EM Sheikh V Rupert A Mendoza D Yang J. Evidence for innate immune system activation in HIV type 1-infected elite controllers. J Infect Dis. (2014) 209:931–9. doi: 10.1093/infdis/jit581, PMID: 24185941 PMC3935475

[29] Bansal A Sterrett S Erdmann N Westfall AO Dionne Odom J Overton ET. Normal T-cell activation in elite controllers with preserved CD4 + T-cell counts. AIDS. (2015) 29:2245–54. doi: 10.1097/QAD.0000000000000860, PMID: 26544698 PMC4773905

[30] Teer E Mukonowenzou NC Essop MF. The role of immunometabolism in HIV-1 pathogenicity: links to immune cell responses. Viruses. (2022) 14:1813. doi: 10.3390/v14081813, PMID: 36016435 PMC9415820

[31] Halliday N Williams C Kennedy A Waters E Pesenacker AM Soskic B. CD86 is a selective CD28 ligand supporting foxP3+ Regulatory T cell homeostasis in the presence of high levels of CTLA-4. Front Immunol. (2020) 11:600000. doi: 10.3389/fimmu.2020.600000, PMID: 33363541 PMC7753196

[32] Köchli C Wendland T Frutig K Grunow R Merlin S Pichler WJ. CD80 and CD86 costimulatory molecules on circulating T cells of HIV infected individuals. Immunol Lett. (1999) 65:197–201. doi: 10.1016/S0165-2478(98)00107-2, PMID: 10065743

[33] Espíndola MS Soares LS Galvao Lima LJ Zambuzi FA Cacemiro MC Brauer VS. HIV infection: focus on the innate immune cells. Immunologic Res. (2016) p:1118–32. doi: 10.1007/s12026-016-8862-2, PMID: 27590022

[34] Naidoo KK Ndumnego OC Ismail N Dong KL Ndung’u T. Antigen presenting cells contribute to persistent immune activation despite antiretroviral therapy initiation during hyperacute HIV-1 infection. Front Immunol. (2021) 12:738743. doi: 10.3389/fimmu.2021.738743, PMID: 34630420 PMC8498034

[35] Thobakgale C Naidoo K McKinnon LR Werner L Samsunder N Karim SA. Interleukin 1-beta (IL-1b) production by innate cells following TLR stimulation correlates with TB recurrence in ART-treated HIV-infected patients. J Acquired Immune Deficiency Syndromes. (2017) 74:213–20. doi: 10.1097/QAI.0000000000001181, PMID: 27654812 PMC5237660

[36] Pinto BF Medeiros NI Teixeira Carvalho A Eloi Santos SM Fontes Cal TC Rocha DA. CD86 expression by monocytes influence an immunomodulatory profile in asymptomatic patients with chronic chagas disease. Front Immunol. (2018) 9:454. doi: 10.3389/fimmu.2018.00454, PMID: 29599775 PMC5857740

[37] Anzinger JJ Butterfield TR Angelovich TA Crowe SM Palmer CS. Monocytes as regulators of inflammation and HIV-related comorbidities during cART. J Immunol Res. (2014) 2014:569819. doi: 10.1155/2014/569819, PMID: 25025081 PMC4082935

[38] Mbonye U Karn J Ari K. The molecular basis for human immunodeficiency virus latency. Annu Rev Virol. (2017) 4:261–85. doi: 10.1146/annurev-virology-101416-041646, PMID: 28715973

[39] Hartana CA Yu XG. Immunological effector mechanisms in HIV-1 elite controllers. Curr Opin HIV AIDS. (2021) 16:243–8. doi: 10.1097/COH.0000000000000693, PMID: 34270465 PMC8373669

[40] Mazzuti L Turriziani O Mezzaroma I. The many faces of immune activation in HIV-1 infection: A multifactorial interconnection. Biomedicines. (2023) 11:159. doi: 10.3390/biomedicines11010159, PMID: 36672667 PMC9856151

[41] Sodora DLS. Guidob, *Immune activation and AIDS pathogenesis* . AIDS. (2008) 22:439–46. doi: 10.1097/QAD.0b013e3282f2dbe7, PMID: 18301056

[42] Vidya Vijayan KK Karthigeyan KP Tripathi SP Hanna LE. Pathophysiology of CD4+ T-Cell depletion in HIV-1 and HIV-2 infections. Front Immunol. (2017) 8:580. doi: 10.3389/fimmu.2017.00580, PMID: 28588579 PMC5440548

[43] Hunt PW Martin JN Sinclair E Bredt B Hagos E Lampiris H. T cell activation is associated with lower CD4+ T cell gains in human immunodeficiency virus-infected patients with sustained viral suppression during antiretroviral therapy. J Infect Dis. (2003) 187:1534–77. doi: 10.1086/374786, PMID: 12721933

[44] Wolint P Betts MR Koup RA Oxenius A. Immediate cytotoxicity but not degranulation distinguishes effector and memory subsets of CD8+ T cells. J Exp Med. (2004) 199:925–36. doi: 10.1084/jem.20031799, PMID: 15051762 PMC2211884

[45] Migueles SA Sabbaghian MS Shupert WL Bettinotti MP Marincola FM Martino L. HLA B* 5701 is highly associated with restriction of virus replication in a subgroup of HIV-infected long term nonprogressors. Proc Natl Acad Sci. (2000) 97:2709–14. doi: 10.1073/pnas.050567397, PMID: 10694578 PMC15994

[46] Pereyra F Addo MM Kaufmann DE Liu Y Miura T Rathod A. Genetic and immunologic heterogeneity among persons who control HIV infection in the absence of therapy. J Infect Dis. (2008) 197:563–71. doi: 10.1086/526786, PMID: 18275276

[47] Vieira VA Millar J Adland E Muenchhoff M Roider J Guash CF. Robust HIV-specific CD4+and CD8+T-cell responses distinguish elite control in adolescents living with HIV from viremic nonprogressors. AIDS. (2022) 36:95–105. doi: 10.1097/QAD.0000000000003078, PMID: 34581306 PMC8654249

[48] Capa L Ayala Suárez R De La Torre Tarazona HE González García J Del Romero J Alcamí J. Elite controllers long-term non progressors present improved survival and slower disease progression. Sci Rep. (2022) 12:16356. doi: 10.1038/s41598-022-19970-3, PMID: 36175445 PMC9522853

[49] Castaño D García LF Rojas M. Increased frequency and cell death of CD16 + monocytes with Mycobacterium tuberculosis infection. Tuberculosis. (2011) 91:348–60. doi: 10.1016/j.tube.2011.04.002, PMID: 21621464

[50] Bonato VL Medeiros AI Lima VM Dias AR Faccioli LH Silva CL. Downmodulation of CD18 and CD86 on macrophages and VLA-4 on lymphocytes in experimental tuberculosis. Scandinavian J Immunol. (2001) 54:564–73. doi: 10.1046/j.1365-3083.2001.00996.x, PMID: 11902331

[51] Kumar V Barrett JE. Toll-like receptors (TLRs) in health and disease: an overview. In: Kumar V, editor. Toll-like Receptors in Health and Disease. Cham: Springer Nature Switzerland (2022). p. 1–21., PMID: 10.1007/164_2021_568 35091824

[52] Chaudhry A Das SR Hussain A Mayor S George A Bal V. The Nef protein of HIV-1 induces loss of cell surface costimulatory molecules CD80 and CD86 in APCs. J Immunol. (2005) 175:4566–74. doi: 10.4049/jimmunol.175.7.4566, PMID: 16177101

[53] Souza PE Rocha MO Menezes CA Coelho JS Chaves AC Gollob KJ. Trypanosoma cruzi infection induces differential modulation of costimulatory molecules and cytokines by monocytes and T cells from patients with indeterminate and cardiac Chagas’ disease. Infection Immun. (2007) 75:1886–94. doi: 10.1128/IAI.01931-06, PMID: 17283096 PMC1865727

[54] Han J Wang B Han N Zhao Y Song C Feng X. CD14(high)CD16(+) rather than CD14(low)CD16(+) monocytes correlate with disease progression in chronic HIV-infected patients. JAIDS J Acquired Immune Deficiency Syndromes. (2009) 52:553–9. doi: 10.1097/QAI.0b013e3181c1d4fe, PMID: 19950429

[55] Funderburg NT Zidar DA Shive C Lioi A Mudd J Musselwhite LW. Shared monocyte subset phenotypes in HIV-1 infection and in uninfected subjects with acute coronary syndrome. Blood J Am Soc Haematology. (2012) 120:4599–608. doi: 10.1182/blood-2012-05-433946, PMID: 23065151 PMC3512236

[56] McCausland MR Juchnowski SM Zidar DA Kuritzkes DR Andrade A Sieg SF. Altered monocyte phenotype in HIV-1 infection tends to normalize with integrase- inhibitor-based antiretroviral therapy. PloS One. (2015) 10:e0139474. doi: 10.1371/journal.pone.0139474, PMID: 26430882 PMC4591977

[57] Campbell JH Hearps AC Martin GE Williams KC Crowe SM. The importance of monocytes and macrophages in HIV pathogenesis,treatment,and cure. AIDS. (2014) 28:2175–87. doi: 10.1097/QAD.0000000000000408, PMID: 25144219 PMC6331181

[58] Thieblemont N Weiss L Sadeghi HM Estcourt C Haeffner‐Cavaillon N. CD14lowCD16high: a cytokine-producing monocyte subset which expands during human immunodeficiency virus infection. Eur J Immunol. (1995) 25:3418–24. doi: 10.1002/eji.1830251232, PMID: 8566032

[59] Varghese M Clemente J Lerner A Abrishami S Islam M Subbaiah P. Monocyte trafficking and polarization contribute to sex differences in meta-inflammation. Front Endocrinol. (2022) 13:826320. doi: 10.3389/fendo.2022.826320, PMID: 35422759 PMC9001155

[60] Rambaran S Maseko TG Lewis L Hassan Moosa R Archary D Ngcapu S. Blood monocyte and dendritic cell profiles among people living with HIV with Mycobacterium tuberculosis co-infection. BMC Immunol. (2023) 24:21. doi: 10.1186/s12865-023-00558-z, PMID: 37480005 PMC10362598

[61] Buitendijk M Eszterhas SK Howell AL. Toll-like receptor agonists are potent inhibitors of human immunodeficiency virus-type 1 replication in peripheral blood mononuclear cells. AIDS Res Hum Retroviruses. (2014) 30:457–67. doi: 10.1089/aid.2013.0199, PMID: 24328502

[62] Leite Pereira A Tchitchek N Lambotte O Le Grand R Cosma A. Characterization of leukocytes from HIV-ART patients using combined cytometric profiles of 72 cell markers. Front Immunol. (2019) 10:1777. doi: 10.3389/fimmu.2019.01777, PMID: 31447833 PMC6691046

[63] Pereira AL Tchitchek N Lopez EM Lambotte O Le Grand R Cosma A. A high-resolution mass cytometry analysis reveals a delay of cytokines production after TLR4 or TLR7/8 engagements in HIV-1 infected humans. Cytokine. (2018) 111:97–105. doi: 10.1016/j.cyto.2018.08.018, PMID: 30138900

[64] Netea MG Nold Petry CA Nold MF Joosten LA Opitz B van der Meer JH. Differential requirement for the activation of the inflammasome for processing and release of IL-1β in monocytes and macrophages. Blood J Am Soc Haematology. (2009) 113:2324–35. doi: 10.1182/blood-2008-03-146720, PMID: 19104081 PMC2652374

[65] Ferrari D Chiozzi P Falzoni S Hanau S Di Virgilio F. Purinergic modulation of interleukin-1β release from microglial cells stimulated with bacterial endotoxin. J Exp Med. (1997) 185:579–82. doi: 10.1084/jem.185.3.579, PMID: 9053458 PMC2196027

[66] Gaidt MM Ebert TS Chauhan D Schmidt T Schmid Burgk JL Rapino F. Human monocytes engage an alternative inflammasome pathway. Immunity. (2016) 44:1097–4180. doi: 10.1016/j.immuni.2016.01.012, PMID: 27037191

[67] Li JZ Segal FP Bosch RJ Lalama CM Roberts Toler C Delagreverie H. Antiretroviral therapy reduces t-cell activation and immune exhaustion markers in human immunodeficiency virus controllers. Clin Infect Dis. (2020) 70:1636–42. doi: 10.1093/cid/ciz442, PMID: 31131858 PMC7146008

[68] Longenecker CT Sullivan C Baker JV. Immune activation and cardiovascular disease in chronic HIV infection. Curr Opin HIV AIDS. (2016) 11:216–25. doi: 10.1097/COH.0000000000000227, PMID: 26599166 PMC4745252

[69] McKibben RA Margolick JB Grinspoon S Li X Palella Jr FJ Kingsley LA. Elevated levels of monocyte activation markers are associated with subclinical atherosclerosis in men with and those without HIV infection. J Infect Dis. (2015) 211:1219–28. doi: 10.1093/infdis/jiu594, PMID: 25362192 PMC4402336

[70] Sereti I Krebs SJ Phanuphak N Fletcher JL Slike B Pinyakorn S. Persistent, Albeit reduced, chronic inflammation in persons starting antiretroviral therapy in acute HIV infection. Clin Infect Dis. (2017) 64:124–31. doi: 10.1093/cid/ciw683, PMID: 27737952 PMC5215214

[71] Crowell TA Gebo KA Blankson JN Korthuis PT Yehia BR Rutstein RM. Hospitalization rates and reasons among HIV elite controllers and persons with medically controlled HIV infection. J Infect Dis. (2015) 211:1692–702. doi: 10.1093/infdis/jiu809, PMID: 25512624 PMC4447832

[72] Sugawara S Reeves RK Jost S. Learning to be elite: lessons from HIV-1 controllers and animal models on trained innate immunity and virus suppression. Front Immunol. (2022) 13:858383. doi: 10.3389/fimmu.2022.858383, PMID: 35572502 PMC9094575

[73] Kwon H Pelletier N DeLuca C Genin P Cisternas S Lin R. Inducible expression of IκBα repressor mutants interferes with NF-κB activity and HIV-1 replication in Jurkat T cells. J Biol Chem. (1998) 273:7431–40. doi: 10.1074/jbc.273.13.7431, PMID: 9516441

