# Supplementary material for: Innate immune dysfunction and persistent activation in South African HIV elite controllers
Source: Front Immunol. 2025 Aug 27;16:1603436. doi: 10.3389/fimmu.2025.1603436 (PMC12420260; doi:10.3389/fimmu.2025.1603436)
Supplement: Supplementary file 1 [file DataSheet1.docx]

**Innate Immune Dysfunction and Persistent Activation in South African HIV Elite Controllers**

Asisipo Mohamed, MSc^1,2^, Yenzekile Zungu, MSc^1,2^, Sharon Shalekoff, PhD^1,2^, Osman Ebrahim, MD, FRCP^3^, Ziyaad Waja, MBChB, MPH^4^, Neil Martinson, MBBCh, MPH^4^, Caroline T. Tiemessen, PhD^1,2^, Christina Thobakgale, PhD^1,2^

^1^School of Pathology, Faculty of Health Science, University of the Witwatersrand, Johannesburg, South Africa.^2^ Centre for HIV and STIs, National Institute for Communicable Diseases, Division of the National Health Laboratory Service, Faculty of Health Sciences, University of the Witwatersrand, Johannesburg, South Africa.^3^ School of Therapeutic Sciences, Department of Pharmacology, Faculty of Health Sciences, University of the Witwatersrand, Johannesburg, South Africa.^4^Perinatal HIV Research Unit, Chris Hani Baragwanath Academic Hospital, University of the Witwatersrand, Soweto, South Africa.

**B**

**A**

**C**

**Supplementary Figure 1**. **Age,** **CD4+ T cell counts and CD4+/CD8+ ratios of study participants. (A)** Age, **(B)** CD4+ T cell count and **(C)** CD4+/CD8+ ratios of PWH_EC_ (n=16), PWH_ART_ (n=18), PWH_PROG_ (n=19_)_, and PWOH_HIV-_ (n=17). Each dot represents an individual, and horizontal lines represent the median with the interquartile range. The Kruskal-Wallis test was used to assess the differences in non-parametric data. Mann-Whitney *U* test was used to assess differences between the respective groups. *P<0.05* was considered statistically significant. Data for three study participants were unavailable.

**FMOs**


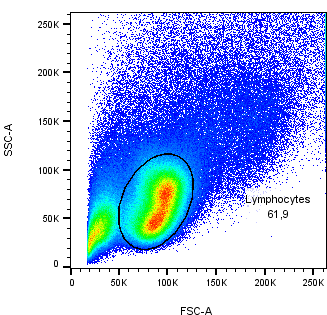

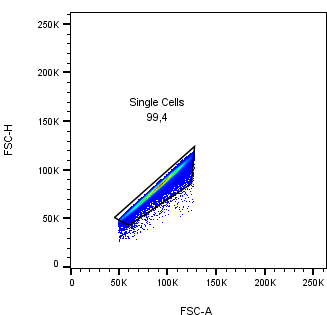

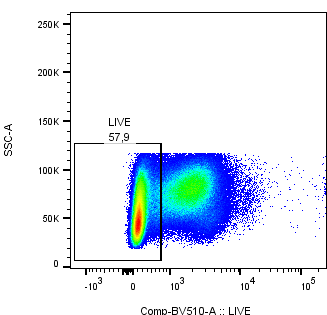

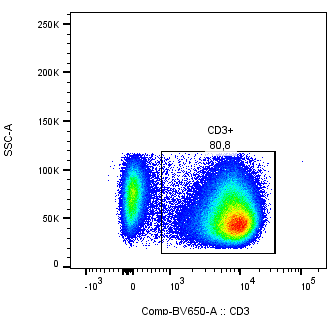

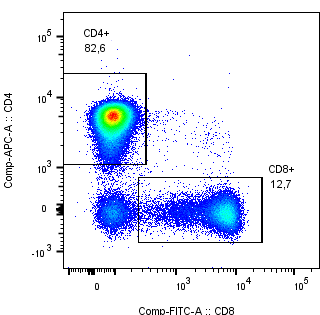


**Single cells**

**Live/Viability**

**Lymphocytes**

**CD3+**

**CD4+/CD8+**


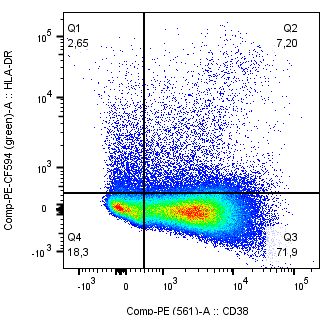


**CD4+ T cell gate**


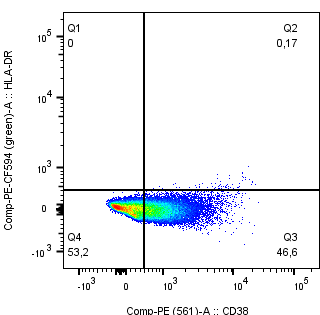

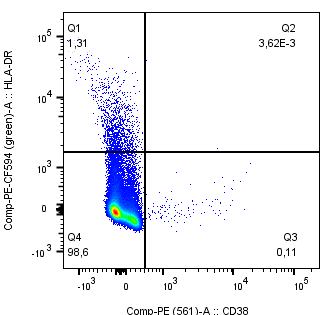


**FMOs**

**HLA-DR FMO**

**CD38 FMO**

**HLA-DR/CD38**


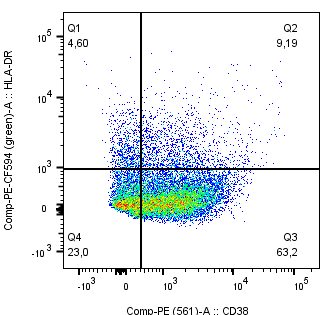

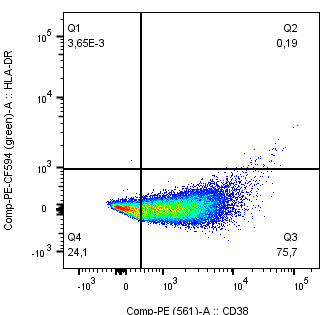

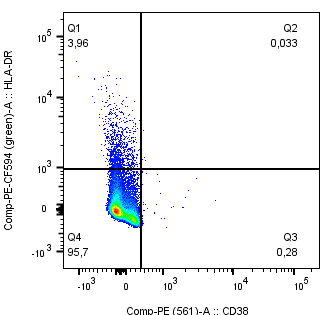


**CD8+ T cell gate**

**HLA-DR FMO**

**CD38 FMO**

**HLA-DR/CD38**

**CD4+ T cells**

**CD8+ T cells**


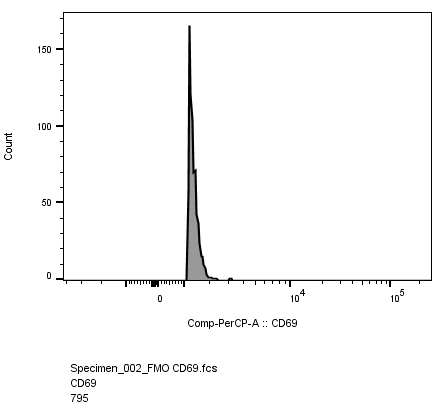

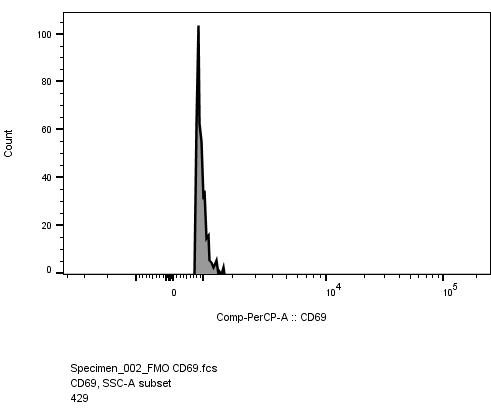

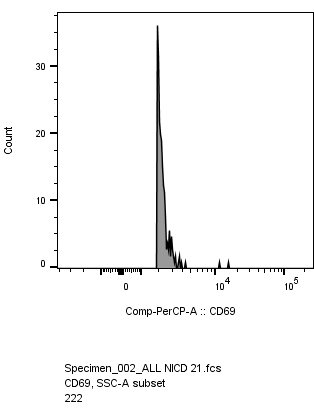

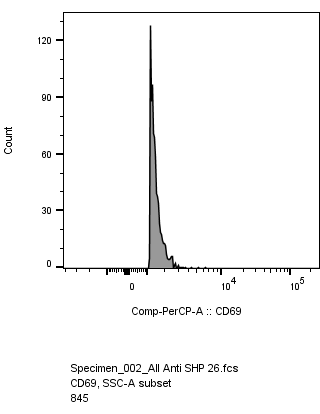

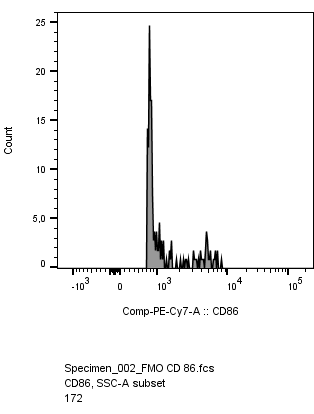

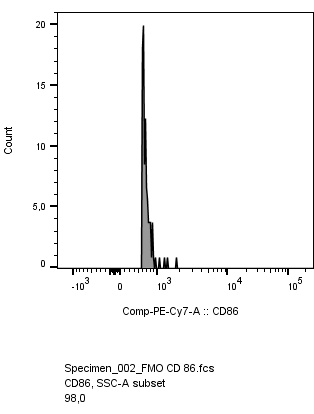

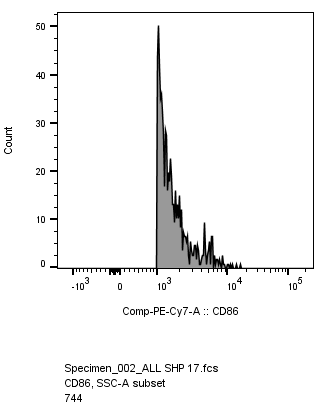

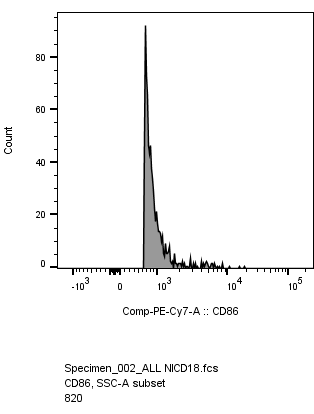

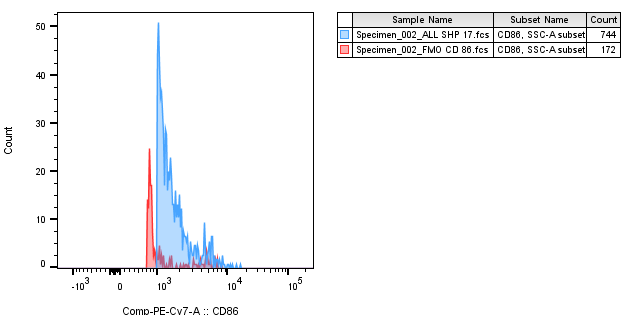

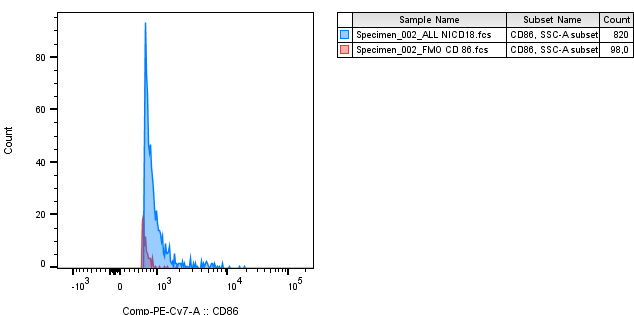

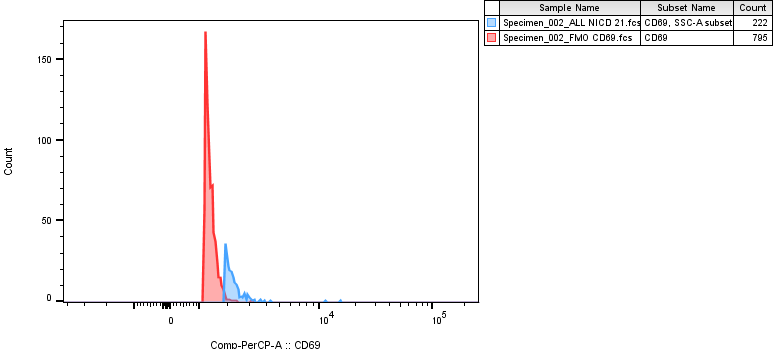

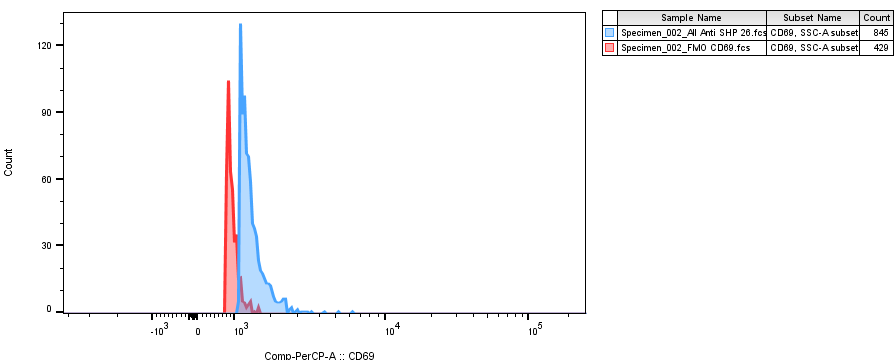


**Supplementary Figure 2. Representative gating strategy for T cell subset activation.** Gating was performed by first selecting lymphocytes, then single cells, and subsequently excluding B cells, NK cells and dead cells. CD3+ T cells were gated from live/viable cells, followed by gating on CD4+ and CD8+ T cells. Activation was measured by the co-expression of HLA-DR+ and CD38+, CD69 and CD86 using fluorescence minus one (FMO) controls to set the gating threshold.

**pDCs and mDCs**

**From HLA-DR +**

**Live/ Viability**

**All cells**

**Single cells**

**HLA-DR+**


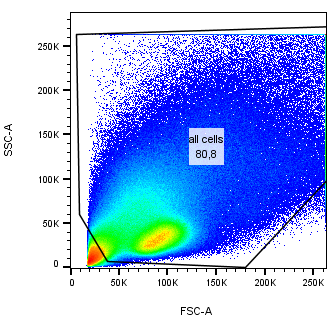


**CD3-**


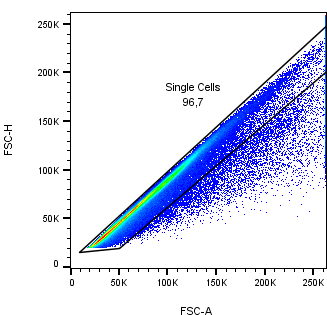

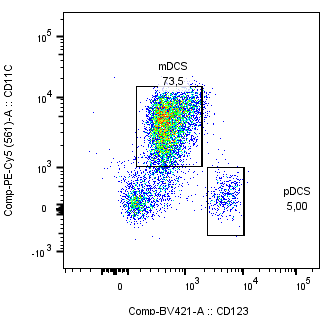

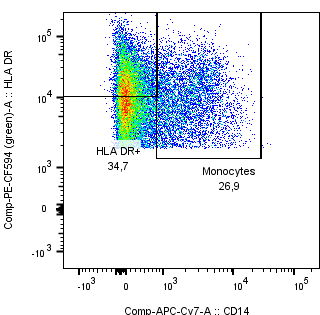

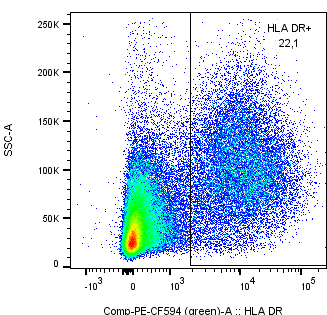

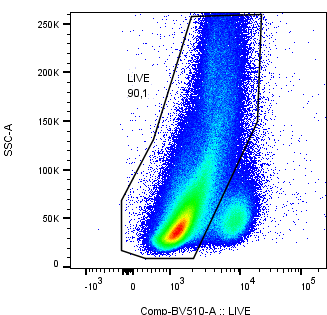

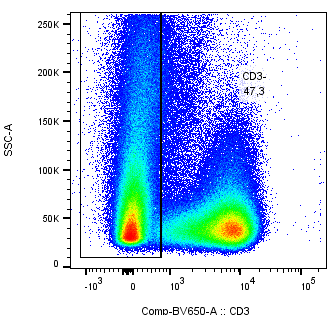


**Supplementary Figure 3. Identification of blood monocyte subsets and antigen-presenting cells.** All cells were initially gated, followed by gating of single cells and successive exclusion of NK cells, B cells, and dead cells. HLA-DR+ cells were gated from the CD3- population. HLA-DR+ CD14+ was gated from HLA-DR+ for monocyte subsets. Monocyte subsets were delineated based on CD14 and CD16 expression: classical (CD14+CD16-), intermediate (CD14++CD16+), inflammatory (CD14dimCD16+) and CD14lowCD16- monocyte subsets. For the identification of dendritic cells (mDCs and pDCs), HLA-DR+CD14- cells were further gated to identify mDCs (CD11c+ CD123-) and pDCs (CD11c-CD123+).

**Unstimulated**


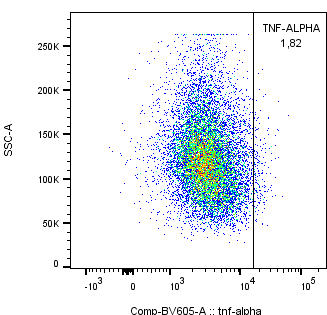

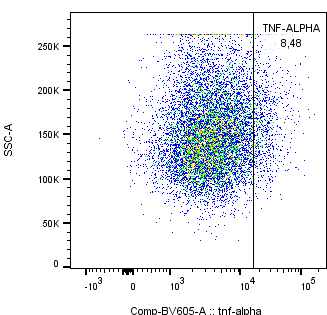

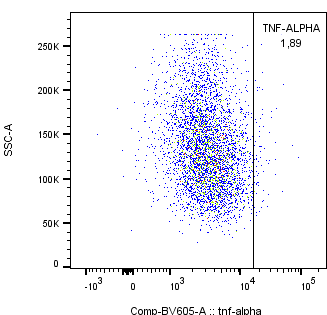

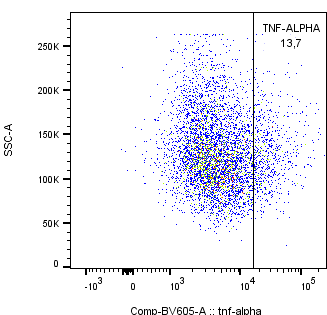

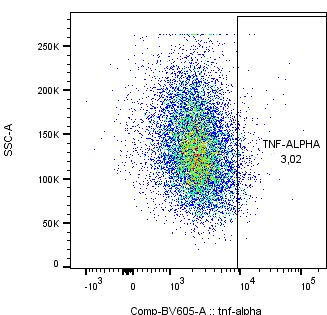

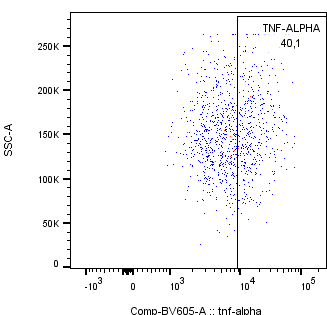

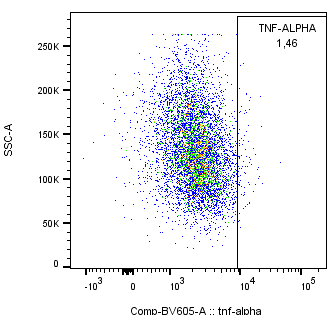

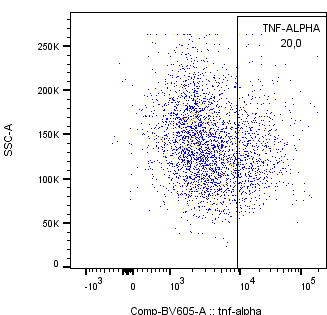

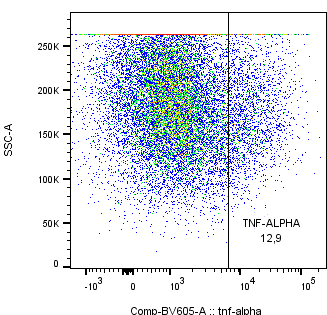

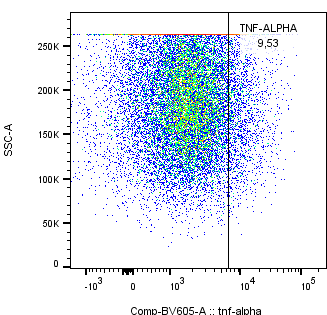

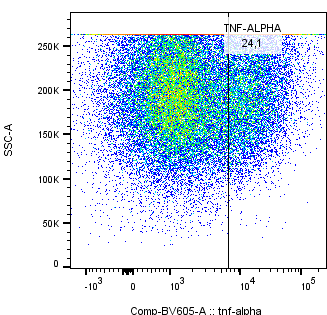

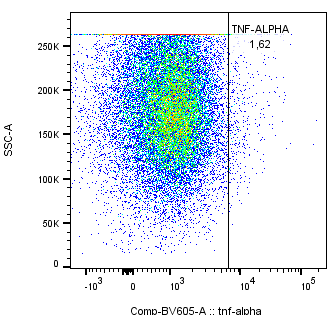

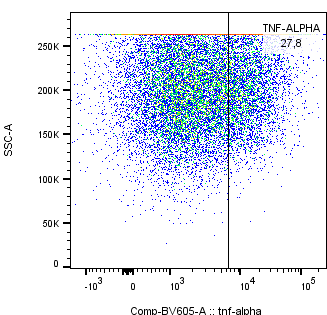

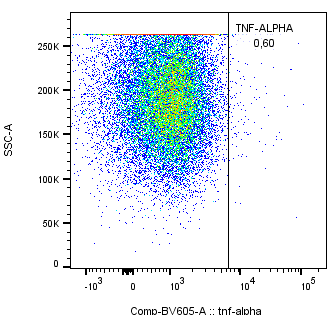

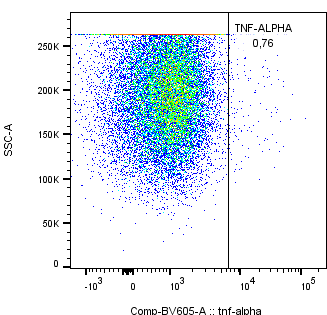

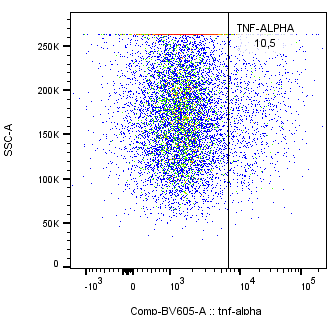


**PWOH_HIV-_**

**TLR4 (LPS)**

**TLR9 (CPG)**

**TLR7/8 (CLO97)**

**PWH_ART_**

**PWH_PROG_**

**PWH_EC_**

**Supplementary Figure 4. Representative gating strategy for cytokine in antigen-presenting cells.** Cytokine expression (TNF-α, IFN-α and IL-1β) was measured in antigen-presenting cells (APCs) of PWOH_HIV-_, PWH_EC_, PWH_PROG_, and PWH_ART_. Following stimulation with Toll-like receptor ligands. **(A)** All cells were gated, followed by single cells, and exclusion of NK, B cells and dead cells. Viable CD3- cells were subsequently gated, followed by gating of HLA-DR+ cells. Within the HLA-DR+CD14+ population, mDCs were identified as CD11c+CD123- and pDCs as CD11c-CD123+ cell subsets. Monocytes, mDCs and pDCs were then assessed for TNF-α, IL-1β and IFN-α production following stimulation with TLR4, TLR7/8 or TLR9 ligands. **(B)** representative example of TNF-α production in mDCs post-stimulation with TLR ligands across the different groups. TNF-α production was reported after unstimulated conditions were used to subtract the background from the stimulated sample.
